# Tyrosine and Phenylalanine Activate Neuronal DNA Repair but Exhibit Opposing Effects on Global Transcription and Adult Female Mice Are Resilient to TyrRS/YARS1 Depletion

**DOI:** 10.1002/iub.70030

**Published:** 2025-06-06

**Authors:** Megha Jhanji, Ashita Bhan, Colin Arrowood, Dina W. Yakout, Ankit Shroff, Danielle McManus, Henrietta Gifford, Janay Vacharasin, Sofia B. Lizarraga, Taras Y. Nazarko, Angela M. Mabb, Mathew Sajish

**Affiliations:** ^1^ Department of Drug Discovery and Biomedical Sciences, College of Pharmacy University of South Carolina Columbia South Carolina USA; ^2^ Department of Biology Georgia State University Atlanta Georgia USA; ^3^ Neuroscience Institute, Center for Behavioral Neuroscience Georgia State University Atlanta Georgia USA; ^4^ Department of Biological Sciences, College of Arts and Sciences University of South Carolina Columbia South Carolina USA; ^5^ Department of Molecular Biology, Cell Biology, and Biochemistry, and Center for Translational Neuroscience Brown University Providence Rhode Island USA

**Keywords:** aminoacyl‐tRNA synthetases, amyloid beta, aromatic amino acids, DNA repair, female resilience, neuronal transcription, resilience to tau accumulation

## Abstract

Serum tyrosine and phenylalanine levels increase during aging and age‐associated disorders. We previously showed that tyrosyl‐tRNA synthetase (TyrRS/YARS1) is reduced in Alzheimer's Disease (AD) brains, and tyrosine and phenylalanine decrease TyrRS in neurons. Here, we found that tau is a negative regulator, whereas estrogen and leucine act as positive regulators of TyrRS. Young female mice exhibit increased TyrRS in the cortex compared to male mice. Notably, young *Tau* knockout male, but not female mice showed increased cortical TyrRS. Tau accumulation in middle‐aged female mice did not decrease cortical TyrRS compared to males, suggesting that middle‐aged females are resilient to tau‐mediated TyrRS depletion. Tyrosine and phenylalanine treatment decreased tubulin tyrosination, activated DNA repair pathways, and protected against etoposide (ETO) and camptothecin (CPT)‐induced toxicity, respectively, in neurons. While tyrosine facilitated topoisomerase 1 (TOP1) recruitment to chromatin and inhibited global transcription, in contrast, phenylalanine recruited topoisomerase 2 beta (TOP2β) to chromatin and stimulated global transcription. Furthermore, tyrosine decreased the presence of DNA fragments in a comet assay whereas phenylalanine increased them. Addition of *cis*‐resveratrol (*cis*‐RSV) protected against tyrosine‐induced transcription inhibition by facilitating the recruitment of both TOP1 and TOP2β to chromatin and increasing tubulin tyrosination. Moreover, *cis*‐RSV decreased both total and phosphorylated tau and protected neurons against amyloid beta (Aβ)‐induced neurite degeneration and DNA damage. Gene expression profiling using human embryonic stem cell (hESC)‐derived neurons demonstrated that *cis*‐RSV is a broad‐spectrum neuroprotective and anti‐viral agent. In contrast, *trans*‐RSV mimics phenylalanine‐induced gene expression, including downregulation of long genes and induction of an AD‐like gene expression signature. This work suggests that age and disease‐associated increases in serum tyrosine and phenylalanine levels would activate neuronal DNA repair while inhibiting transcription and tubulin tyrosination. *cis*‐RSV protects against their toxicity by restoring tubulin tyrosination, TOP1 and TOP2β‐mediated transcription, and decreasing tau in primary neurons.

## 1 Introduction

Tyrosine and phenylalanine were generated under primitive earth conditions [1]. During evolution, tyrosine and phenylalanine were commonly substituted for each other because of their similar physicochemical properties and encoding codons [2]. However, during the evolution of metazoans, tyrosine phosphorylation emerged as a regulator of major signaling pathways [3], potentially triggering a selective pressure for tyrosine depletion within the proteome of multicellular organisms along with the addition of tyrosine phosphatase and spatiotemporal/contextual regulation of tyrosine kinases [4]. While metazoans evolved to accommodate numerous tyrosine kinases encoded in their genomes, in contrast, the genomes of the unicellular yeasts *Schizosaccharomyces pombe* and *Saccharomyces cerevisiae* do not encode tyrosine kinases, and the expression of Src tyrosine kinase is toxic in yeast, presumably due to deleterious tyrosine phosphorylation [5, 6]. However, whether the loss of tyrosine residues from proteins in metazoans [4] was driven by positive selection to remove deleterious phosphorylation sites remains controversial [7, 8, 9]. Moreover, drug resistant cancer cells exploit codon‐biased translation reprogramming to increase the incorporation of tyrosine in proteins upregulated in the rewired proteome [10]. Since tyrosine accumulation is toxic to the cells [11, 12, 13], it is reasonable that metazoans would have evolved multiple mechanisms to manage tyrosine accumulation while accommodating selective evolutionary pressure to deplete tyrosine residues from their proteome.

Microtubules (MTs) are part of cytoskeletal components, which are conserved among all eukaryotic species and are involved in intracellular transport, organelle positioning, cell shape, and cell motility [14]. Microtubules are assembled from heterodimers of α‐tubulin and β‐tubulin [14], which are heterogeneous in length and highly dynamic, undergoing rapid stochastic polymerization and depolymerization cycles. This “dynamic instability” property [15] allows microtubules to reorganize and adapt to specific cellular contexts and contributes significantly to their physiological functions [14]. In eukaryotic cells, the C terminus of α‐tubulin undergoes a cycle of detyrosination–retyrosination reaction, in which the C‐terminal tyrosine or phenylalanine residue of αtubulin is cleaved by tubulin carboxypeptidases/detyrosinases [14] and re‐added to the chain by the tubulin‐tyrosine ligase (TTL) [16]. Removal of the penultimate glutamate residue results in α‐tubulin (delta2 tubulin) that cannot be tyrosinated. Neurons [11] and cardiomyocytes [17] are striking examples of cells sensitive to tyrosine accumulation, with microtubules being essential for supporting morphological and functional complexity [18]. Microtubule‐associated protein tau (MAPT) is an endogenous regulator of tubulin tyrosination in the brain [19] and heart [20]. Interestingly, amyloid beta (Aβ) is an inhibitor of tubulin tyrosination [21, 22] and tau is essential for Aβ‐induced neurotoxicity [19, 21, 23, 24]. Consistently, tau depletion [19, 24, 25, 26] and activators of tubulin tyrosination [22] protect against Aβ‐induced neurotoxicity.

Transcription inhibition is a hallmark of aging [27, 28], especially for long genes [28, 29]. Neurons express some of the longest‐known genes, which are downregulated during aging [28, 29] and in Alzheimer's disease (AD) [30, 31]. During transcription, topoisomerase I (TOP1) and TOP2 cut supercoiled DNA to introduce single‐strand breaks (SSBs) and double‐strand DNA breaks (DSBs), respectively, and covalently bind to one of the nicked DNA ends to allow the other nicked DNA to pass through the intact DNA in a controlled manner. After rotation, TOP1 and TOP2 re‐ligate the SSBs [32] and DSBs [33] respectively. DSBs induced by topoisomerase 2 beta (TOP2β) can also facilitate the transcription of highly expressed genes [34, 35], and TOP1 can prevent transcription overactivation [36]. While TOP1 and TOP2β can promote the expression of long genes [37, 38], TOP1 is essential for transcription associated with normal synaptic transmission [39] and neuronal survival [40], and TOP2β facilitates activity‐dependent gene expression in neurons [35]. Camptothecin (CPT) is a natural compound that prevents the re‐ligation activity of TOP1 [41], which can inhibit transcription [42] especially of genes associated with inflammation [43]. While etoposide (ETO) inhibits the re‐ligation activity of TOP2, in contrast, it activates transcription associated with inflammation in neurons [44]. DNA methylation on cytosine (5 mC) of CpG islands in gene promoters represses transcription, but SSBs activate cytosine demethylation in neurons [45]. Ten‐eleven translocation methylcytosine dioxygenases (TETs) induced hydroxylation of methylated DNA (5mC– > 5hmC) [46] is part of the DNA repair process [47], which removes 5mC to alleviate gene repression. Therefore, 5‐hmC accumulation exhibits a regulatory role in transcription [48].

Although we previously showed that tyrosine induces oxidative DNA damage [11], which inhibits transcription [49, 50], whether tyrosine acquires a physiological function in age‐associated transcription inhibition [27, 28] remains unknown. Interestingly, inhibitors of both protein synthesis and transcription protect against DNA damage‐induced apoptosis [51], and tyrosine and leucine have opposite effects on protein synthesis [52]. While higher serum leucine protects against heart failure [53, 54] and cognitive dysfunction [55, 56], in contrast, higher serum tyrosine exacerbates heart failure [17] and cognitive dysfunction in the oldest adults [57]. Recent works indicated that elevated serum tyrosine/phenylalanine promotes heart failure and neurodegeneration by depleting nuclear TyrRS in cardiomyocytes [17] and neurons [11], respectively. Most importantly, brain TyrRS levels correlate positively with cognition and memory [11] and women generally exhibit better memory and processing speed than men in middle age [58]. Since this female advantage diminishes with age [58], and serum tyrosine increases during aging [11] and is lower in young females [11], whether decreased serum tyrosine in young females would lead to a compensatory increase in TyrRS in the female brain remains unknown.

Here, we found that the addition of tyrosine inhibited global transcription, tubulin tyrosination, and activated the DNA damage response (DDR) in rat cortical neurons. Tyrosine addition also protected cortical neurons against the TOP2β selective inhibitor, etoposide. In contrast, the addition of phenylalanine increased global transcription and SSB repair, which protected cortical neurons against TOP1 inhibitor‐induced neurotoxicity. Further, we found that estrogen increases neuronal TyrRS, as young female mice exhibited higher TyrRS levels than young males in cortical neurons. We also found that while leucine is a positive regulator, tau is a negative regulator of TyrRS in primary cortical neurons and male mouse cortex. However, tau accumulation in middle‐aged female mice did not decrease cortical TyrRS, suggesting that middle‐aged female mice are resilient to tau‐induced TyrRS depletion potentially due to female sex‐specific molecular pathways [59, 60] that activate global protein synthesis [61, 62]. Interestingly, *cis*‐resveratrol (*cis*‐RSV), which protects against tyrosine‐mediated neurotoxicity [11] rescued tyrosine‐mediated transcription inhibition, 5 hmC/5 mC accumulation, and DNA repair, and Aβ‐induced neurite degeneration and DNA damage accumulation potentially by activating tubulin tyrosination with concomitant depletion of tau in cortical neurons, providing novel mechanistic insights into the neuroprotective actions of *cis*‐RSV.

## 2 Results

### 2.1 Tyrosine and Phenylalanine Have Opposing Effects on Global Transcription in Cortical Neurons

**FIGURE 1:**
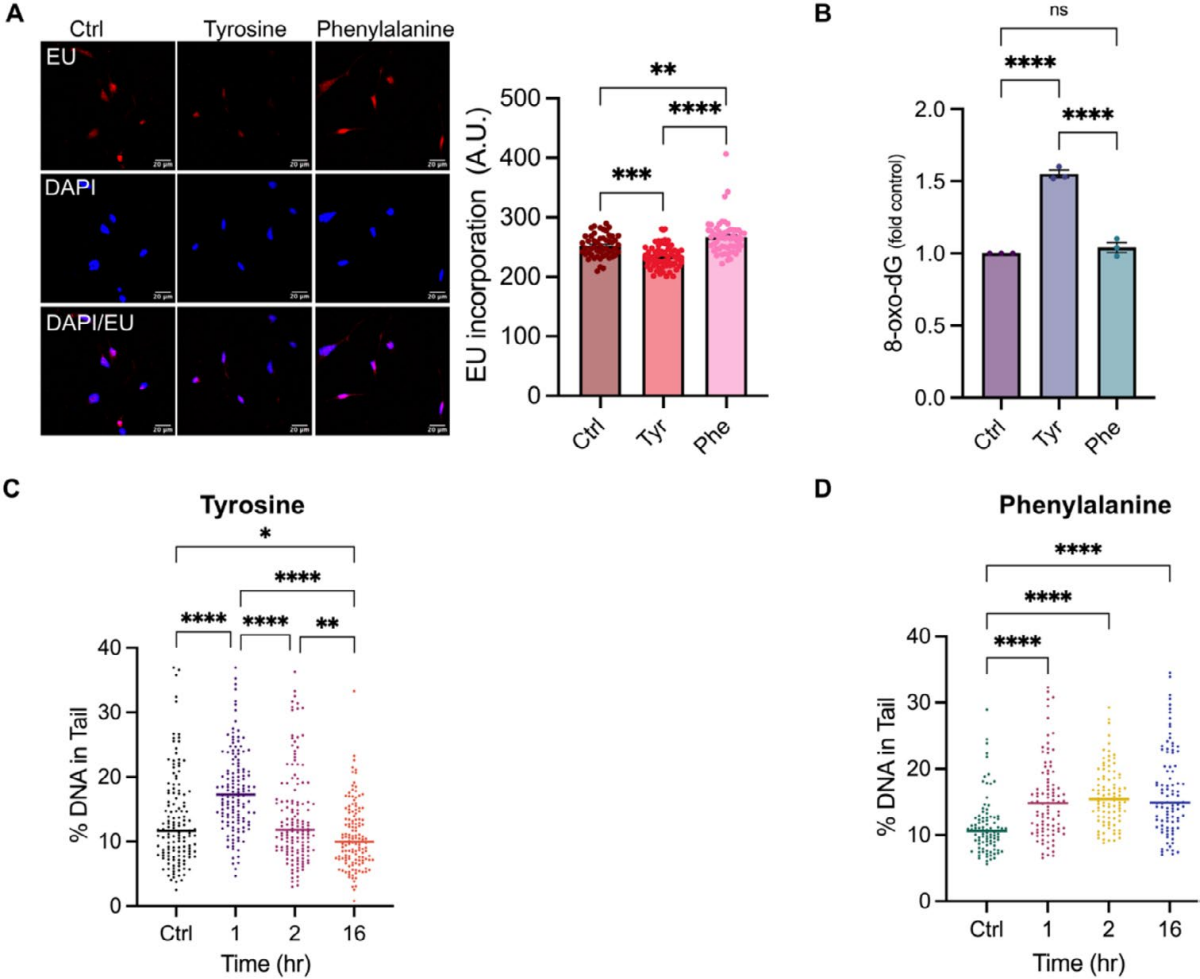
Tyrosine and phenylalanine exhibit opposing effects on global transcription and DNA repair in neurons. (A) Tyrosine and phenylalanine have differential effects on new RNA synthesis in neurons. Quantitative IF analysis of EU incorporation in nascent RNA in primary cortical neurons treated with tyrosine (500 μM) or phenylalanine (500 μM) for 4 h, followed by the addition of EU for 30 min and subsequent processing for image analysis as per the manufacturer's protocol (*n* = 20 neurons per condition from *N* = 3 independent experiments). (B) Tyrosine and phenylalanine have differential effects on oxidative DNA damage accumulation. Quantification of 8‐oxo‐2′‐dG in primary cortical neurons (DIV9/10) after 16 h treatment with tyrosine (500 μM) or phenylalanine (500 μM) (*n* = 30 neurons per condition from *N* = 3 independent experiments). (C) Tyrosine activates DNA repair. Quantification of DNA in comet tails from primary cortical neurons (DIV 9/10) treated with tyrosine (500 μM) for up to 16 h (*n* ≥ 100 nuclei for *N* = 3 independent experiments). (D) Phenylalanine sustains DNA damage. Quantification of DNA in comet tails from primary cortical neurons (DIV 9/10) treated with phenylalanine (500 μM) for up to 16 h (*n* ≥ 100 nuclei for *N* = 3 independent experiments). Statistical significance was determined using two‐way ANOVA with Tukey's multiple comparisons test. Data are presented as mean ± SEM from three independent experiments, and *p* values (* ≤ 0.05, ** ≤ 0.01, *** ≤ 0.001, and **** ≤ 0.0001) are indicated in the figures. Statistical significance was determined using two‐way ANOVA with Tukey's multiple comparisons test.

We previously showed that tyrosine induces oxidative DNA damage [11], which inhibits transcription [49, 50]. Although serum tyrosine increases during aging [11], whether tyrosine acquired any physiological function in transcription contributing to age‐associated transcription inhibition [27, 28] remains unknown. Phenylalanine did not cause neurotoxicity in our previous analysis [11]. However, we noticed that plasma phenylalanine also increases during aging [63, 64] and causes cardiac aging [65, 66], suggesting a critical role of phenylalanine in age‐associated disorders. To determine the effects of tyrosine and phenylalanine on global transcription, we treated rat cortical neurons (DIV 9/10) with tyrosine or phenylalanine for 4 h, followed by a 30‐min labeling using the nucleoside analog 5‐ethynyl uridine (EU) and quantified the extent of EU incorporation in nascent RNA. We found that while tyrosine significantly reduced EU incorporation, in contrast, phenylalanine increased EU incorporation (Figure 1A). Since oxidative stress can also inhibit transcription [49, 50] and tyrosine is known to induce oxidative stress [11], we quantified the levels of 8‐oxo‐2′‐deoxyguanosine (8‐oxo‐dG) levels, one of the major products of DNA oxidation. While tyrosine increased 8‐oxo‐dG levels, phenylalanine had no significant effect (Figure 1B), providing another potential molecular basis for tyrosine‐mediated transcription inhibition. Transcription is the major source of DNA damage in neurons [35, 67] and conversely, transcription inhibition triggers DNA repair [49, 68]. Therefore, we determined the effect of tyrosine and phenylalanine on neuronal DNA damage response (DDR) using an alkaline comet assay in a time‐dependent manner. We observed that tyrosine induced DNA damage in 1 h, but it was rapidly repaired in 2 h (Figure 1C). Additionally, at 16 h, neurons treated with tyrosine showed a reduced amount of DNA fragments in the comets compared to control (Figure 1C). Conversely, neurons treated with phenylalanine exhibited sustained presence of DNA fragments in the comets compared to both control and tyrosine‐treated neurons (Figure 1D). Together, these results suggest that while elevated phenylalanine levels may inhibit DNA repair, potentially due to an increase in global transcription, sustained increase in tyrosine levels may enhance DNA repair, potentially by inhibiting global transcription.

### 2.2 Tyrosine and Phenylalanine Distinctly Regulate the Recruitment of TOP1 and TOP2β to Chromatin

**FIGURE 2:**
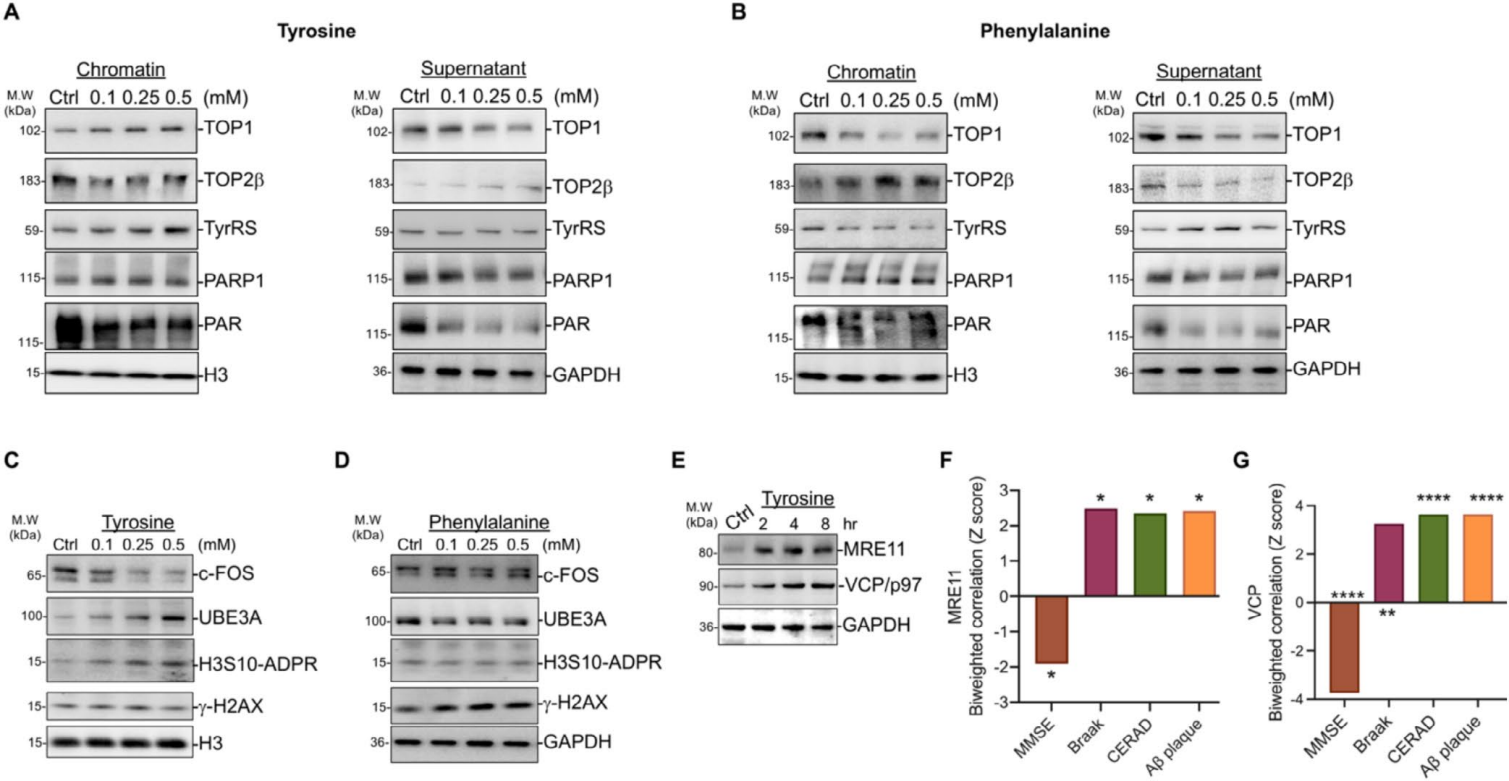
Tyrosine and phenylalanine differentially regulate TOP1 and TOP2β‐mediated transcriptional activities. (A) Tyrosine facilitates the recruitment of TOP1 to chromatin. Rat cortical neurons (DIV 9/10) were treated with tyrosine (0.1–0.5 mM) for 1 h, and chromatin‐associated proteins were isolated using centrifugation. The presence of TOP1, TOP2β, PARP1, PARylation, TyrRS, and histone H3 or GAPDH in the pellet and supernatant fractions, respectively, was detected by WB using their corresponding antibodies. (B) Phenylalanine promotes TOP2β recruitment to chromatin. Rat cortical neurons (DIV 9/10) were treated with phenylalanine (0.1–0.5 mM) for 1 h, followed by WB analysis of chromatin‐associated proteins as in (A). (C) Tyrosine increases UBE3A and H3‐Ser‐ADPR levels. Rat cortical neurons (DIV 9/10) were treated with tyrosine (0.1–0.5 mM) for 4 h, and WB detected changes in c‐FOS, UBE3A, H3‐Ser‐ADPR, and γ‐H2AX. (D) Phenylalanine increases γ‐H2AX levels. Rat cortical neurons (DIV 9/10) were treated with phenylalanine (0.1–0.5 mM) for 4 h, followed by WB analysis for c‐FOS, UBE3A, H3‐Ser‐ADPR, and γ‐H2AX. (E) Tyrosine increases DSB repair factors. Rat cortical neurons (DIV 9/10) were treated with tyrosine (0.5 mM) for up to 8 h, and the changes in MRE11 and VCP/p97 levels were detected by WB using their corresponding antibodies. (F and G) Brain MRE11 (F) and VCP (G) levels negatively correlate with cognition and positively correlate with AD progression. Graphs showing the correlation between MRE11 (F) and VCP/p97 (G) with cognitive performance and AD progression from re‐analyzed AD brain proteome data. MMSE indicates cognitive performance score, whereas Braak and CERAD scores indicate AD progression (*p* values * ≤ 0.05, ** ≤ 0.01, *** ≤ 0.001, and **** ≤ 0.0001).

While TOP1‐induced SSBs can inhibit the transcription of many genes [36], TOP2β‐induced DSBs can facilitate the transcription of many genes [34, 35]. Given the opposite effects of tyrosine and phenylalanine on global transcription (Figure 1A), we hypothesized that these amino acids would differentially affect the recruitment of TOP1 and TOP2β to chromatin. To investigate this, we treated rat cortical neurons (DIV 9/10) with increasing concentrations of tyrosine or phenylalanine for 1 h. Following treatment, the cell lysates were centrifuged to separate chromatin‐bound proteins from unbound proteins, allowing us to assess the recruitment of TOP1 and TOP2β. We found that tyrosine increased TOP1 and TyrRS levels while decreasing TOP2β in the chromatin fraction (Figure 2A), suggesting that tyrosine‐mediated nuclear depletion of TyrRS does not occur at 1 h. In contrast, phenylalanine decreased TOP1 and TyrRS and recruited TOP2β to the chromatin (Figure 2B). However, both tyrosine and phenylalanine recruited poly‐ADP‐ribose polymerase 1 (PARP1) to chromatin, indicating that both treatments induced DNA damage (Figure 2A,B). TOP1 regulates the expression of ubiquitin‐protein ligase E3A (UBE3A) [69], while TOP2β‐mediated DSBs trigger the induction of c‐FOS [35]. To assess the effects of tyrosine and phenylalanine on UBE3A and c‐FOS induction, we determined the changes in UBE3A and c‐FOS levels in cortical neurons (DIV 9/10) treated with tyrosine and phenylalanine for 4 h. Tyrosine treatment increased UBE3A levels while decreasing c‐FOS levels (Figure 2C) whereas phenylalanine treatment decreased UBE3A while increasing c‐FOS (Figure 2D). TOP1‐induced DNA breaks “trap” PARP1 [70], which triggers ADP‐ribosylation of the serine side chains of histone H3 (H3‐Ser‐ADPR) [71] whereas DSBs are strong inducers of phosphorylation of the histone variant H2A.X on serine 139 (p‐H2AX or γ‐H2AX) [72]. Consistently, tyrosine increased H3‐Ser‐ADPR levels without affecting γ‐H2AX levels, whereas phenylalanine decreased H3‐Ser‐ADPR levels while increasing γ‐H2AX levels (Figure 2C,D). Together, these results indicate that tyrosine and phenylalanine distinctly regulate TOP1 and TOP2β‐induced DNA breaks and transcriptional activities in cortical neurons. Valosin‐containing protein (VCP) or transitional endoplasmic reticulum ATPase also known as p97 (VCP/p97) [73] and MRE11 [74] are essential for the repair of TOP2/ETO‐induced DSBs. To explore the mechanism of tyrosine‐mediated reduction in the amount of DNA fragments in the comets compared to control at 16 h (Figure 1C), we determined the levels of MRE11 and VCP/p97 after treatment with tyrosine for up to 8 h in rat cortical neurons (DIV 9/10). We found that tyrosine increased MRE11 and VCP/p97 levels (Figure 2E), providing a potential molecular basis for tyrosine‐mediated activation of DSB repair. Furthermore, supporting a potential role of elevated tyrosine in AD development [11] and cognitive dysfunction in the oldest adults [57], our re‐analysis of the human brain proteome [75] showed that brain MRE11 and VCP levels correlate negatively with cognition and memory scores and positively with the standard measures of AD progression (Figure 2F,G).

### 2.3 Both Tyrosine and Phenylalanine Stimulate DNA Synthesis‐Associated Repair (SAR) in Neurons

**FIGURE 3:**
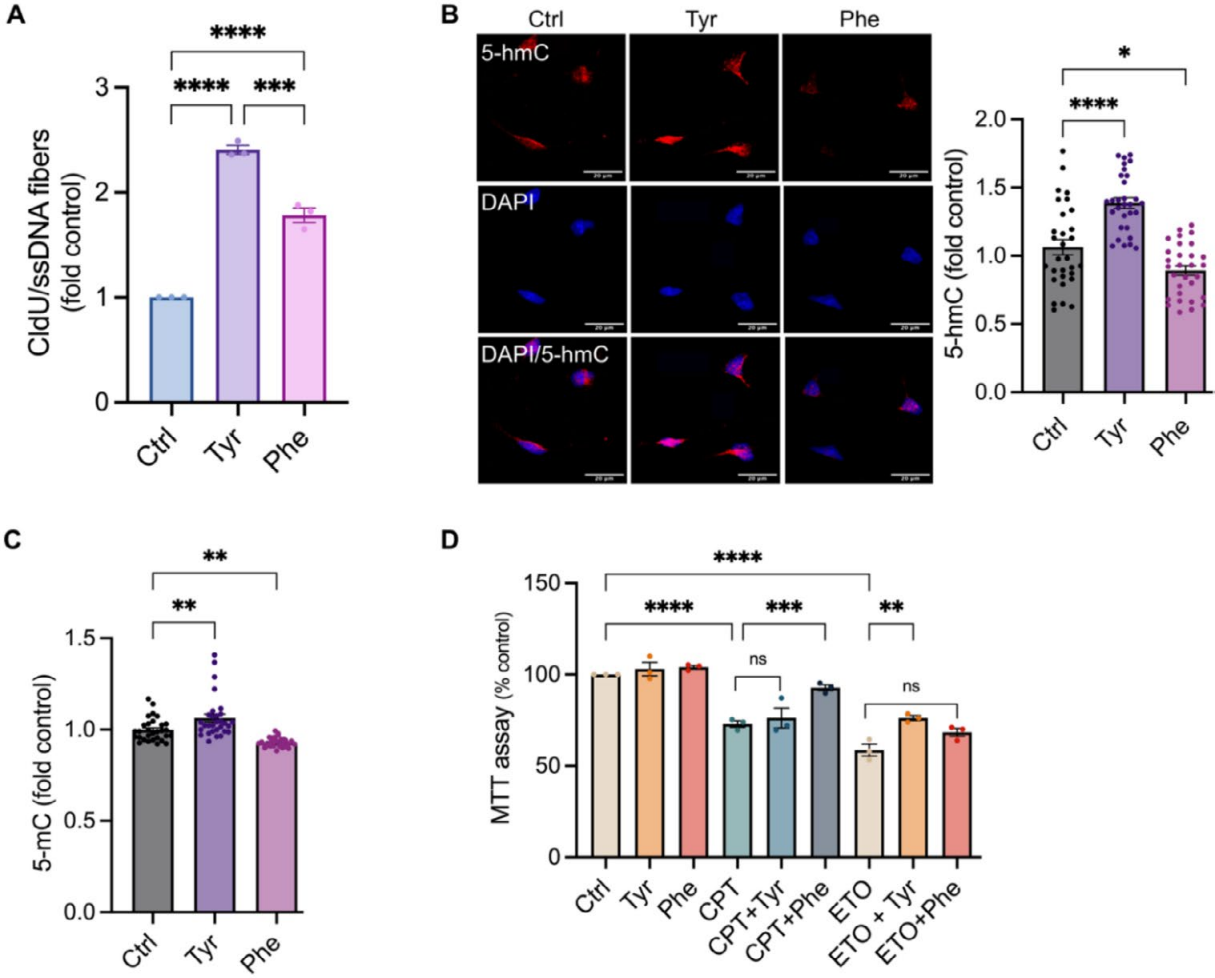
Tyrosine and phenylalanine stimulate DNA synthesis‐associated repair (SAR) and protect against DNA damage‐induced toxicity in neurons. (A) Tyrosine and phenylalanine increase the incorporation of nucleotides in the genomic DNA of cortical neurons. Primary cortical neurons (DIV 9/10) were treated with tyrosine (500 μM) or phenylalanine (500 μM) for 4 h followed by a 30 min pulse labeling using 50 μM deoxynucleoside analog, CldU (5‐chloro‐2′‐deoxyuridine). DNA fiber assay was performed as described in the Methods. (*n* = 20 images per condition from *N* = 3 independent experiments). Statistical significance was determined using two‐way ANOVA with Tukey's multiple comparisons test. (B and C) Tyrosine and phenylalanine have opposing effects on neuronal 5‐hmC/5‐mC levels. Quantitative immunofluorescent (IF) analysis of the changes in the 5‐hmC (B) and 5‐mC (C) in rat cortical neurons treated with tyrosine (500 μM) or phenylalanine (500 μM) for 4 h. (*n* = 30 neurons per condition from *N* = 3 independent experiments). Statistical significance was determined using two‐way ANOVA with Tukey's multiple comparisons test. (D) Tyrosine and phenylalanine have differential effects on CPT and ETO‐induced neurotoxicity. Rat cortical neurons were treated with CPT (10 μM) or ETO (10 μM) either alone or in combination with tyrosine (500 μM) or phenylalanine (500 μM) for 24 h and quantified for neuronal survival using MTT assay, *n* = 3. Statistical significance was determined using two‐way ANOVA with Sidak's multiple comparisons test. Data are presented as mean ± SEM from three independent experiments and *p* values are indicated in the figures (* ≤ 0.05, ** ≤ 0.01, *** ≤ 0.001, and **** ≤ 0.0001).

DNA synthesis‐associated repair (SAR) occurs at SSBs and DSBs sites in neurons [45, 76], and SAR accompanies active DNA demethylation [45, 77]. We previously showed that treatment with *cis*‐RSV and *trans*‐RSV inhibited SAR in cortical neurons [11]. However, only *cis*‐RSV protected against tyrosine‐induced neurodegeneration [11] and *trans*‐RSV caused neurodegeneration [11], potentially due to nucleotide depletion [78]. To determine whether tyrosine and phenylalanine would affect SAR, we treated rat cortical neurons (DIV 9/10) with tyrosine or phenylalanine for 4 h, followed by a 30 min pulse labeling using the deoxynucleoside analog, CldU (5‐chloro‐2′‐deoxyuridine) and subjected them to DNA fiber assay as we reported previously [11]. We found that both tyrosine and phenylalanine increased CldU incorporation into DNA, although tyrosine had a significantly larger effect (Figure 3A). To determine the impacts of tyrosine and phenylalanine on DNA demethylation, we treated rat cortical neurons (DIV 9/10) with tyrosine or phenylalanine for 4 h and quantified 5‐hmC and 5‐mC. We found that while tyrosine increased, in contrast, phenylalanine decreased both 5‐hmC and 5‐mC (Figure 3B,C), suggesting distinct effects of tyrosine and phenylalanine on SAR‐mediated DNA demethylation. Since SAR occurs at both SSB and DSB sites [45, 76], and phenylalanine decreased TOP1 in the chromatin fraction (Figure 2B) and tyrosine increased VCP and MRE11 levels (Figure 2E), we next determined the effect of tyrosine and phenylalanine treatment on CPT or ETO‐induced neurotoxicity. While tyrosine protected against ETO, it did not show any effect against CPT‐induced neurotoxicity (Figure 3D). In contrast, phenylalanine protected against CPT but not against ETO‐induced neurotoxicity (Figure 3D). Together, these results indicate distinct effects of tyrosine and phenylalanine on neuronal SSB and DSB repair and epigenetic regulation through DNA methylation and expression of DNA repair factors. Since neuronal activity‐induced DNA damage and associated transcription are essential for memory formation [35, 39, 76, 79, 80], together, these results suggest that tyrosine and phenylalanine‐mediated activation of neuronal DNA repair might contribute to AD development.

### 2.4 Tau and Detyrosinated Tubulin Are Negative Regulators of TyrRS in Primary Neurons

**FIGURE 4:**
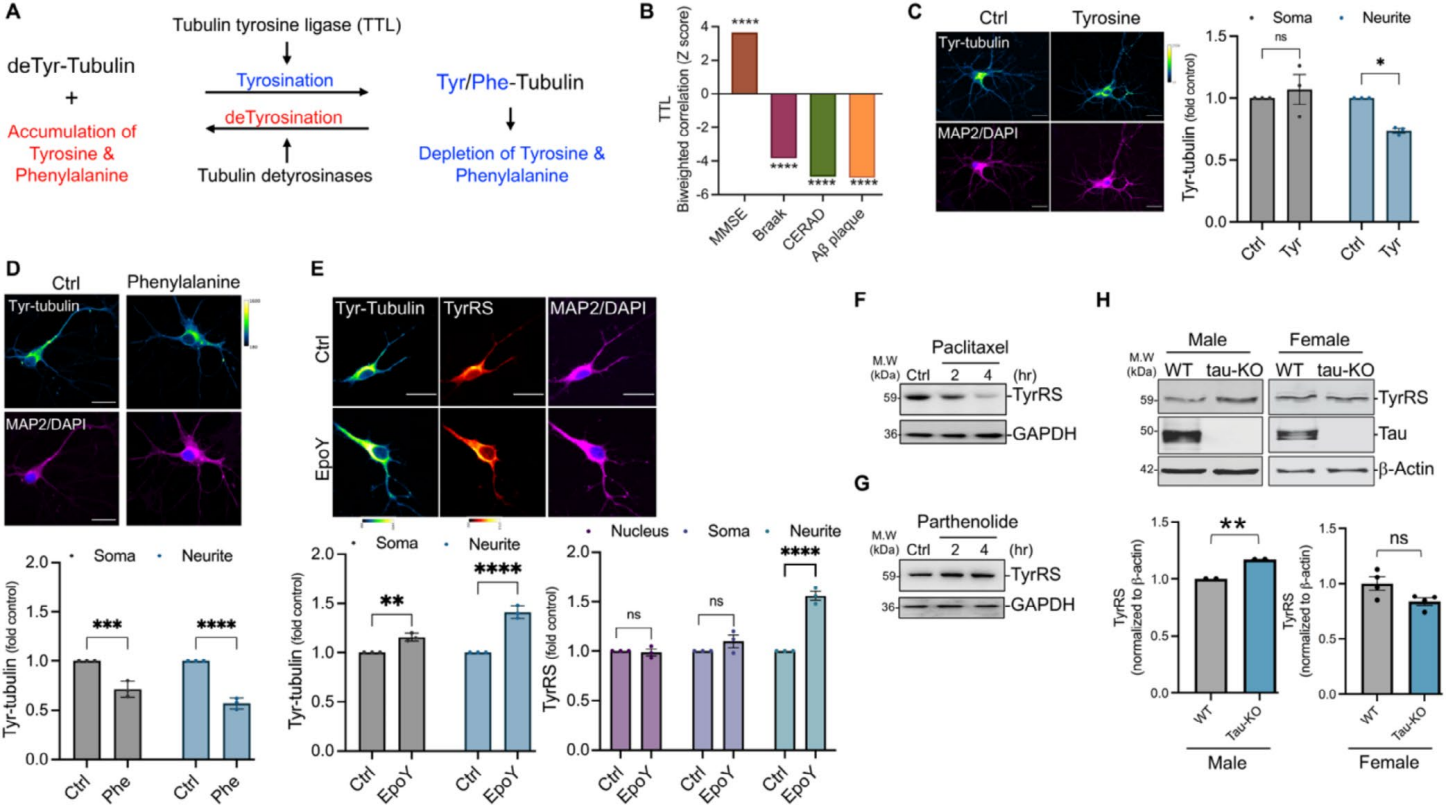
Tau and modulators of the tubulin tyrosination/detyrosination cycle influence TyrRS in neurons. (A) Schematic of the tubulin tyrosination/detyrosination (Tyr/deTyr) cycle. (B) Brain TTL levels correlate positively with cognition and memory. Graph indicating the correlation between TTL and cognitive performance/AD progression from re‐analyzed publicly available AD brain proteome data. (C) Tyrosine decreases tubulin tyrosination (Tyr‐tubulin) in the neurites. Spectral images and quantitative IF of Tyr‐tubulin in primary cortical neurons (DIV 9/10) after tyrosine (500 μM) treatment for 4 h. (*n* = 30 neurons per condition from *N* = 3 independent experiments). Statistical significance was determined using two‐way ANOVA with Šídák's multiple comparisons test. (D) Phenylalanine decreases tubulin tyrosination in both soma and neurites. Spectral images and quantitative IF of Tyr‐tubulin in primary cortical neurons (DIV 9/10) after phenylalanine (500 μM) treatment for 4 h. (*n* = 30 neurons per condition from *N* = 3 independent experiments) Statistical significance was determined using two‐way ANOVA with Šídák's multiple comparisons test. (E) Inhibition of tubulin detyrosination increases neuronal TyrRS levels. Spectral images and quantitative IF of TyrRS in primary cortical neurons (DIV 9/10) treated with EpoY (10 μM) for 2 h. (*n* = 30 neurons per condition from *N* = 3 independent experiments). Statistical significance was determined using two‐way ANOVA with Šídák's multiple comparisons test for Tyr‐tubulin and Tukey's multiple comparison test for TyrRS. (F) Paclitaxel decreases neuronal TyrRS. WB analysis of TyrRS in primary cortical neurons treated with paclitaxel (10 nM) for up to 4 h. (G) Parthenolide increases neuronal TyrRS. WB analysis of TyrRS in primary cortical neurons treated with parthenolide (10 μM) for up to 4 h. (H) Tau KO cortices exhibit increased TyrRS in male mice. WB analysis of TyrRS in cortices from WT and Tau KO male and female mice. Data represent average fold change and standard error for normalized TyrRS for two batches of male mice, and average and standard error for normalized TyrRS in female mice (*n* = 4). Other data represent mean ± SEM from *n* = 3 independent experiments with *p* values indicated in the figures (* ≤ 0.05, ** ≤ 0.01, *** ≤ 0.001, and **** ≤ 0.0001).

We recently showed that both tyrosine and phenylalanine decrease neuronal TyrRS [11]. Since tubulin detyrosination/re‐tyrosination is a major regulator of cellular tyrosine and phenylalanine (Figure 4A), we hypothesized that modulators of tubulin tyrosination would affect neuronal TyrRS. Notably, our reanalysis of the AD brain proteome data showed that similar to TyrRS, brain TTL levels correlate positively with cognition and memory scores and negatively with the standard measures of AD progression (Figure 4B). To determine if tyrosine or phenylalanine would affect neurite tubulin tyrosination (Tyr‐tubulin), we treated rat cortical neurons (DIV 9/10) with tyrosine and phenylalanine. We found that while tyrosine selectively decreased Tyr‐tubulin only in the neurites (Figure 4C), phenylalanine decreased Tyr‐tubulin in the soma and in the neurites (Figure 4D). To explore the relationship between neuronal TyrRS and Tyr‐tubulin, we treated rat cortical neurons (DIV 9/10) with EpoY, a specific inhibitor of VASH1/2‐SVBP tubulin carboxypeptidases [81] and found that EpoY increased TyrRS (Figure 4E). Similarly, treatment with paclitaxel that stabilizes microtubules through detyrosination [82] depleted TyrRS (Figure 4F), and in contrast, treatment with parthenolide that inhibits tubulin detyrosination [83] increased neuronal TyrRS (Figure 4G). Together, these results show that microtubule dynamics can be a major regulator of TyrRS in primary neurons.

Tau negatively regulates tubulin tyrosination in the brain [19] and heart [20], and therefore, we further explored the role of tau in regulating neuronal TyrRS. We recently reported that tau depletes Arc protein in a proteasome‐dependent but ubiquitin‐independent manner [84]. Tau selectively binds to tRNAs [85] and inhibits neuronal protein synthesis [86, 87, 88, 89] and memory formation [90, 91]. Brain‐derived neurotrophic factor (BDNF) stimulates tubulin tyrosination [92], and tau inhibits both BDNF expression [93] and tubulin tyrosination [19]. Given that tau negatively regulates BDNF induction, inhibits neuronal protein synthesis, and tubulin tyrosination in the brain [19] and heart [20], we hypothesized that tau is a negative regulator of neuronal TyrRS. To test this, we measured TyrRS in cortical extracts prepared from *Tau* knockout mice (KO). We found an increase in TyrRS in *Tau* KO neurons from males (Figure 4H). In contrast, we observed a non‐significant decrease in TyrRS in female *Tau* KO neurons (Figure 4H), suggesting a sex‐specific effect of tau on neuronal TyrRS. Together, these data indicate that modulators of tubulin tyrosination and sex‐specific changes in tau are potent regulators of TyrRS in primary neurons.

### 2.5 Estrogen and Leucine Increase TyrRS in Neurons and Adult Female Mice Are Resilient to Tau‐Mediated TyrRS Depletion

**FIGURE 5:**
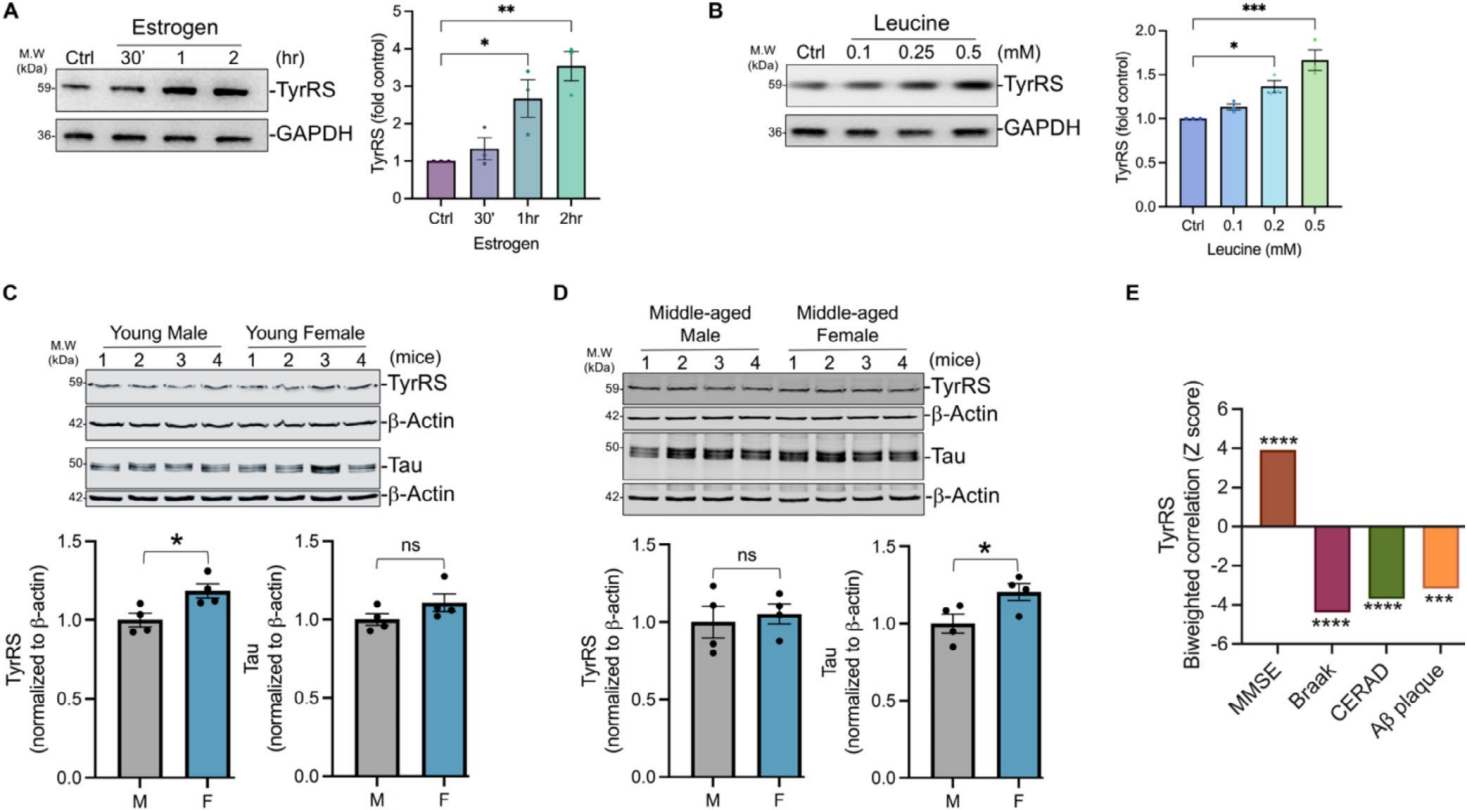
Young female mice exhibit higher cortical TyrRS protein levels than males, and middle‐aged female mice are resilient to tau‐mediated TyrRS depletion, and estrogen and leucine increase TyrRS in neurons. (A) Estrogen increases neuronal TyrRS. Representative western blot (WB) and quantification showing the time‐dependent TyrRS accumulation in rat cortical neurons (DIV 9/10) after estrogen (10 nM) treatment, *n* = 3. (B) Leucine increases neuronal TyrRS. Representative WB and quantification showing the dose‐dependent TyrRS accumulation in rat cortical neurons (DIV 9/10) after leucine (0.1–0.5 mM) treatment, *n* = 3. (C) Young female mice exhibit higher TyrRS levels in the cortex than young male mice. WB image and quantification of TyrRS and tau in the cortices of 4‐month‐old female and male mice. Data represent average and standard error, *n* = 4. (D) Middle‐aged female mice are resilient to tau‐mediated cortical TyrRS depletion. WB image and quantification of TyrRS and tau in the cortices of 12‐month‐old female and male mice. Data represent average and standard error, *n* = 4. The experiment was performed with 3 independent batches of mice. Shown are the results for a representative batch. (E) Human brain TyrRS correlates positively with cognition and memory. Graph showing the correlation of TyrRS with cognitive performance and AD progression based on re‐analysis of publicly available AD brain proteome data as described in the Methods. MMSE indicates cognitive performance score whereas Braak and CERAD scores indicate AD progression. (A, B) Data are presented as mean ± SEM from three independent experiments. Statistical significance was determined using two‐way ANOVA with Tukey's multiple comparisons test, and *p* values are indicated in the figures (* ≤ 0.05, ** ≤ 0.01, *** ≤ 0.001, and **** ≤ 0.0001).

Females exhibit higher levels of tau due to the expression of an X‐linked ubiquitin specific peptidase 11 (USP11), which deubiquitinates tau to prevent its degradation [94] and activates global protein synthesis [61]. Estrogen stimulates USP11 expression [95] and tau protects neurons against oxidative stress [96, 97]. Female mice are resilient to both amyloid beta [98]‐ and tau [99]‐mediated cognitive decline and comorbidities [100]. Further supporting the importance of USP11 in cognition and memory, our reanalysis of the human brain proteome showed that brain USP11 levels correlate positively with cognition and memory scores and negatively with standard measures of AD progression (Figure S1A). Notably, post‐menopausal women are at increased risk of developing dementia (including AD) than men [101, 102], which is thought to be due to a longer lifespan (4–5 years) in women [103]. Although females accumulate AD pathology at faster rates than males [104], they better preserve brain structure compared to males with similar levels of tau pathology [105]. These observations suggest that females exhibit higher resilience to the detrimental effects of tau and AD pathology [59], potentially mediated through activation of female sex‐specific molecular pathways [60]. However, molecular mechanisms underlying these sex‐dependent associations between AD pathology and cognition remain unclear [59, 60]. Although tau binds to tRNAs [85] and inhibits neuronal protein synthesis [86, 87, 88, 89], estrogen stimulates the expression of BDNF [106, 107] and activates neuronal protein synthesis [62]. While inhibitors of protein synthesis decrease neuronal TyrRS [11], BDNF and activators of protein synthesis increase its levels [11]. Based on these observations, we hypothesized that estrogen‐dependent BDNF expression and global protein synthesis activation would contribute to increased TyrRS protein in young female mice neurons. To test our hypothesis, we treated cortical neurons DIV (9/10) with estrogen. Quantification of the changes in TyrRS showed that estrogen is a potent stimulator of neuronal TyrRS (Figure 5A). Branched‐chain amino acids (BCAAs) are also potent stimulators of estrogen receptor alpha (ERα) [108] that overcome metabolic dysfunctions associated with ovariectomy [109]. Moreover, tyrosine and leucine have opposite effects on protein synthesis [52]. Therefore, to determine if L‐leucine would mimic the effects of estrogen on neuronal TyrRS, we treated rat cortical neurons (DIV 9/10) with L‐leucine. Quantification of the changes in TyrRS showed that L‐leucine is also a potent stimulator of neuronal TyrRS (Figure 5B).

We noticed that deficits in recall after contextual fear memory were not observed in young 4‐month‐old AD female model mice [98] but emerged significantly at 8 months of age and later, while in the corresponding male mice, recall deficits were seen from 2 to 12 months of age [98]. To determine whether there are sex‐specific differences in neuronal TyrRS, we measured TyrRS from cortical extracts from young/adult male and female mice (4‐month‐old). Western blot analysis showed that, compared to male mice, young/adult female mice had increased TyrRS but not tau (Figure 5C). This female‐specific increase in TyrRS was not observed at middle age (12‐month‐old) (Figure 5D). However, middle‐aged females had significantly higher accumulation of tau compared to males (Figure 5D), suggesting that middle‐aged females might be resilient to tau‐mediated TyrRS depletion, perhaps due to activation of female sex‐specific molecular pathways [59, 60] that activate global protein synthesis [61, 62]. Further supporting the importance of TyrRS in cognition and memory, our reanalysis of the human brain proteome showed that brain TyrRS levels correlate positively with cognition and memory scores, and negatively with standard measures of AD progression (Figure 5E).

### 2.6 *Cis*‐Resveratrol Decreases Tau and Increases Tubulin Tyrosination in Cortical Neurons

**FIGURE 6:**
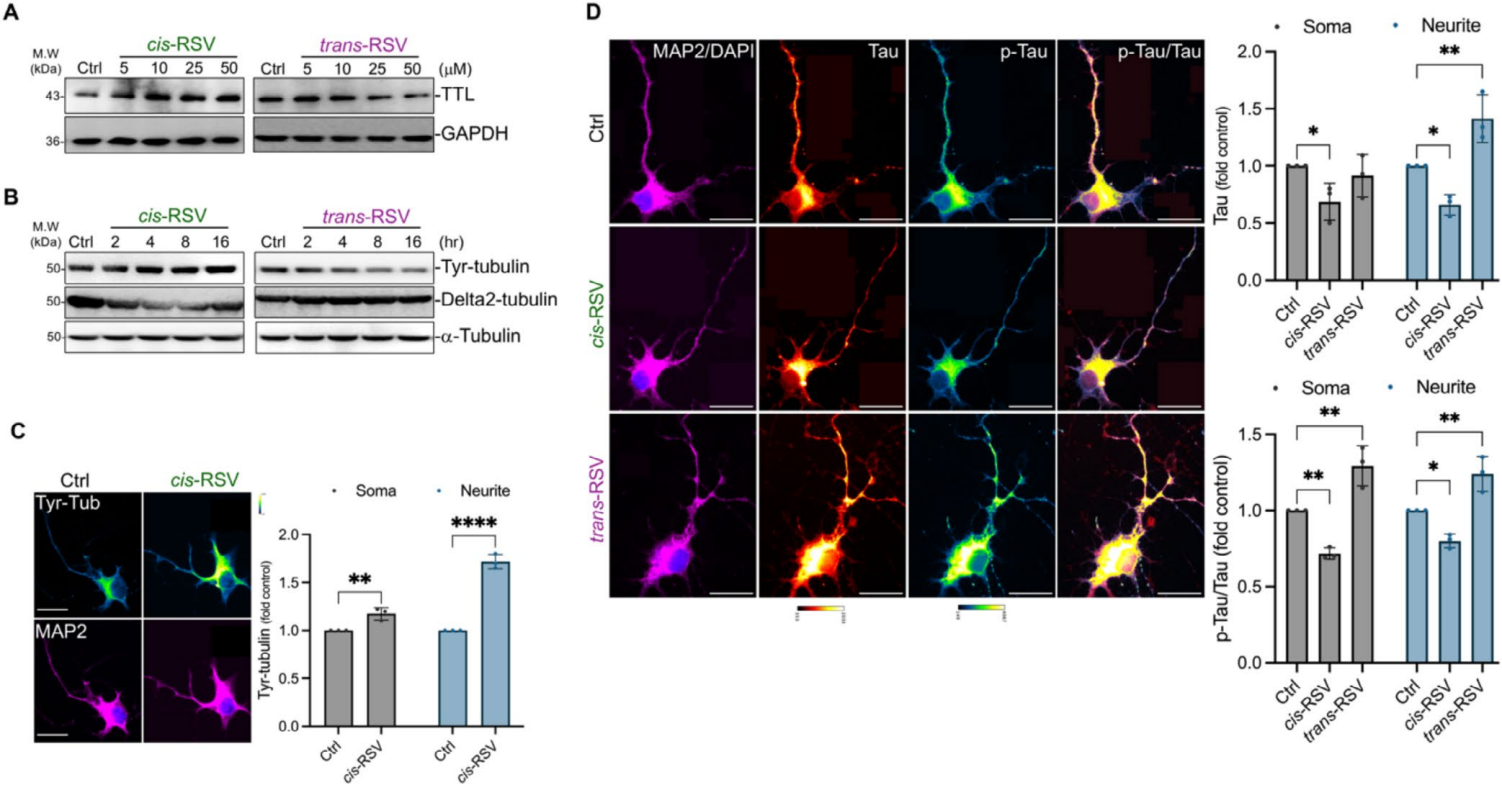
*cis*‐Resveratrol increases tubulin tyrosination and reduces tau and its phosphorylation in neurons. (A) *cis*‐RSV and *trans*‐RSV have opposing effects on neuronal TTL. Representative WB images of TTL in primary cortical neurons (DIV 9/10) treated with *cis*‐RSV and *trans*‐RSV (5–50 μM) for 8 h. (B) *cis*‐RSV and *trans*‐RSV have opposing effects on neuronal tubulin tyrosination and delta2‐tubulin. Representative WB images of Tyr‐tubulin and delta2‐tubulin in primary cortical neurons (DIV 9/10) treated with *cis*‐RSV and *trans*‐RSV (50 μM) for up to 16 h. (C) *cis*‐RSV increases tubulin tyrosination (Tyr‐Tub) in both soma and neurites. Spectral images and quantitative IF analysis of Tyr‐Tub in rat cortical neurons (DIV 9/10) after treatment with *cis*‐RSV (50 μM) for 4 h. (*n* = 30 neurons per condition for *N* = 3 independent experiments) (D) *cis‐RSV* and *trans*‐RSV exert opposite effects on total tau and its phosphorylation status in neurons. Spectral images and quantitative IF analysis of tau and p‐tau in the soma and neurites of rat cortical neurons (DIV 9/10) after treatment with *cis*‐RSV and *trans*‐RSV (50 μM) for 4 h (*n* = 30 neurons for *n* = 3 independent experiments). Data are presented as mean ± SEM from three independent experiments. Statistical significance was determined using two‐way ANOVA with Tukey's multiple comparisons test, and *p* values are indicated in the figures (* ≤ 0.05, ** ≤ 0.01, *** ≤ 0.001, and **** ≤ 0.0001).

We recently showed that *cis*‐RSV and *trans*‐RSV have opposite effects on neuronal protein synthesis and TyrRS [11]. However, the mechanism by which *trans*‐RSV inhibits neuronal protein synthesis [11] remains unclear. Given increased TyrRS in the cortices of *Tau* KO mice (Figure 4H), and the fact that tau binds to tRNAs [85] and inhibits neuronal protein synthesis [86, 87, 88, 89] and tubulin tyrosination [19], we hypothesized that *cis*‐RSV and *trans*‐RSV would affect neuronal tau and tubulin tyrosination levels differently. To determine if cis‐RSV and *trans*‐RSV would affect Tyr‐tubulin levels, we treated rat cortical neurons (DIV 9/10) with *cis*‐RSV and *trans*‐RSV for up to 16 h. Consistent with our previous work [11], *cis*‐RSV and *trans*‐RSV exhibited opposite effects on TTL, tubulin tyrosination, and delta2 tubulin levels (Figure 6A,B). *Cis*‐RSV increased Tyr‐tubulin levels in both soma and the neurites (Figure 6C). To determine if cis‐RSV and *trans*‐RSV would affect tau levels and its phosphorylation (p‐tau) status, we treated rat cortical neurons (DIV 9/10) with *cis*‐RSV and *trans*‐RSV. Treatment with *cis*‐RSV decreased tau in the soma and neurites and also decreased its phosphorylation (p‐tau) (Figure 6D). In contrast, *trans*‐RSV increased p‐tau in both soma and neurites while increasing total tau only in the neurites (Figure 6D). Together, these results suggest that cis‐nd *trans*‐RSV may exploit the deTyr/re‐Tyr‐tubulin cycle and tau to regulate TyrRS and protein synthesis in primary neurons.

### 2.7 *Cis*‐Resveratrol Protects Against Aβ‐Mediated TyrRS Depletion and Neurite Degeneration

Aβ stimulates tubulin detyrosination [21, 22] and tau is a critical mediator of Aβ‐induced toxicity at the postsynapse [23], which is associated with neurodegeneration [19, 21, 24]. Since tau depletion [19, 24, 25, 26] and activators of tubulin tyrosination [22] protect against Aβ‐mediated neurotoxicity, and *cis*‐RSV and *trans*‐RSV exhibited differential effects on tubulin tyrosination and tau levels (Figure 6A–D), we quantified changes in TyrRS and neurite degeneration in rat cortical neurons after treatment with neurotoxic Aβ peptide 1–42 (Aβ_42_) in a time‐and dose‐dependent manner either alone or in combination with *cis*‐RSV and *trans*‐RSV. Towards this purpose, we prepared synthetic Aβ_42_ based on previously published methods [110, 111] and treated rat cortical neurons (DIV9/10) with 50 nanomolar Aβ_42_ (nMAβ_42_) for 24 h. We quantified TyrRS using WB and found that nMAβ_42_ decreased TyrRS in a time‐ and dose‐dependent manner (Figure S1B,C). However, nMAβ_42_ did not affect PheRSβ levels (Figure S1B), indicating a specific effect of nMAβ_42_ on neuronal TyrRS. In contrast to Aβ_42_, its reverse peptide (42‐1) did not affect TyrRS (Figure S1D). We previously showed that *cis*‐RSV bound TyrRS facilitates protein phosphatase 2A (PP2A)‐dependent dephosphorylation of eukaryotic elongation factor 2 (eEF2) [11], suggesting a critical role of TyrRS in the elongation step of protein synthesis as well. Further supporting nMAβ_42_‐induced TyrRS depletion (Figure S1B,C), treatment with nMAβ_42_ increased phosphorylation of eEF2 (Figure S1E), providing a potential molecular basis for nMAβ_42_‐induced TyrRS depletion via neuronal protein synthesis inhibition and deficits in recall after contextual fear memory in 8‐to 12‐month‐old AD female model mice [98].

**FIGURE 7:**
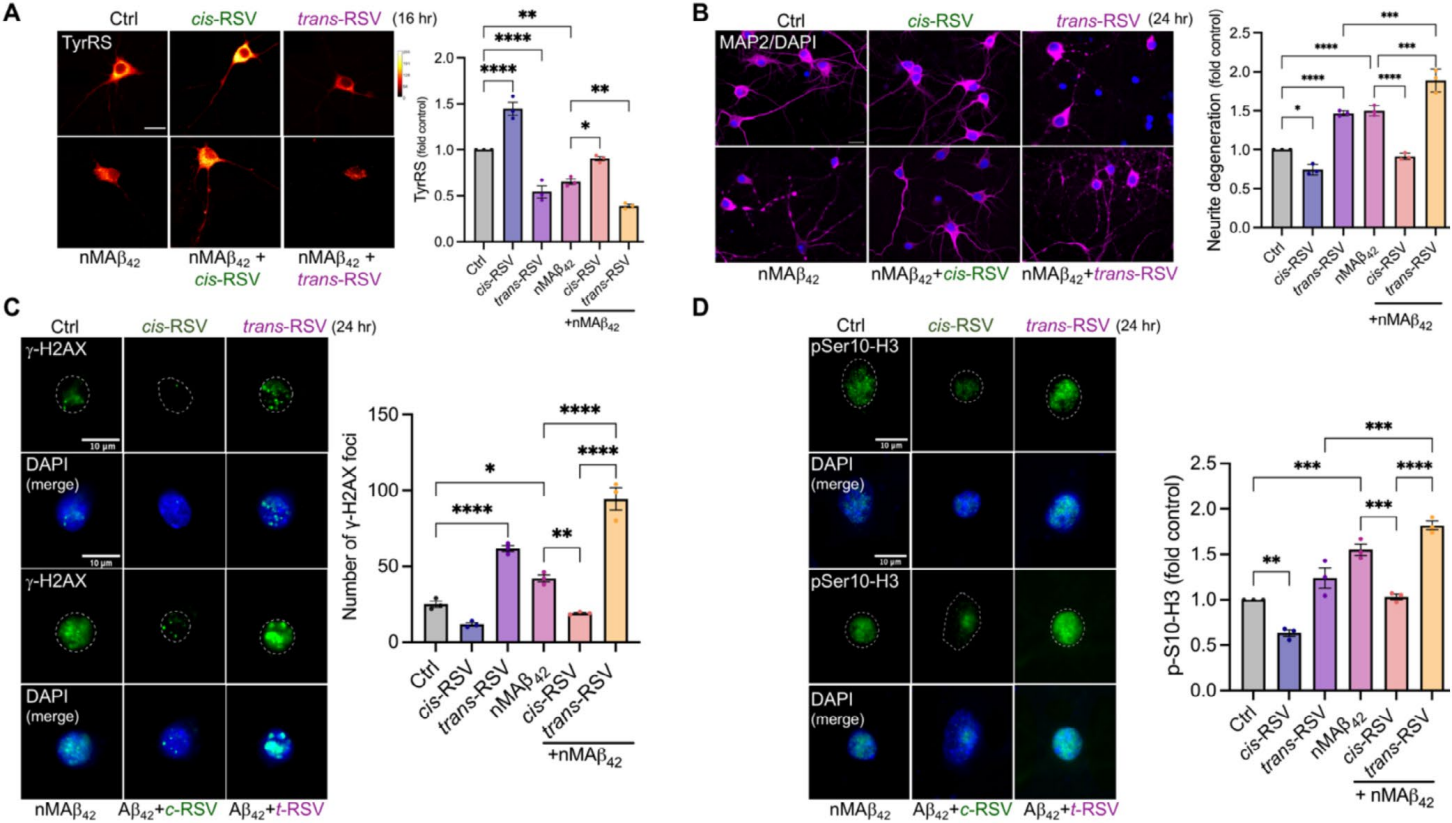
*cis*‐RSV and *trans*‐RSV have opposite effects on Aβ‐induced neurite degeneration and DNA damage response. (A) *cis‐*RSV and *trans*‐RSV have opposite effects on Aβ‐induced TyrRS depletion. Representative spectral images (scale bar, 20 μm) and quantification of TyrRS protein in rat cortical neurons (DIV 10) treated with *cis*‐RSV or *trans*‐RSV, alone or in combination with nMAβ_42_ (50 nM) for 16 h. (B) *cis*‐RSV prevents, while trans‐RSV exacerbates nMAβ42‐induced neurite degeneration. Representative images (scale bar, 20 μm) of cortical neurons pre‐treated with *cis*‐RSV and *trans*‐RSV (50 μM) for 16 h, followed by nMAβ_42_ (50 nM) exposure for 24 h. Neurons were stained with MAP2 (neurite marker, magenta) and DAPI (nuclear marker, blue). Neurons were immunoassayed with anti‐MAP2 antibody and quantified for neurite degeneration (*n* = 15 images per condition with *N* = 3 independent experiments). (C) *cis*‐RSV and *trans*‐RSV have opposite effects on nMAβ42‐mediated DNA damage accumulation. Immunostaining images (scale bar, 10 μm) for DNA damage marker, pSer139‐H2AX foci (γ‐H2AX, green; DAPI—nuclear marker, blue) in cortical neurons (DIV 10) treated with *cis*‐RSV and *trans*‐RSV (50 μM) alone or in combination with *nMAβ*_*42*_ (50 nM) for 24 h. The graph represents the average number of γ‐H2AX foci per nuclei for *n* = 30 neurons per treatment condition for *n* = 3 independent experiments. (D) *cis‐*RSV and *trans*‐RSV have opposite effects on nMAβ42‐induced histone H3 phosphorylation. Immunostaining images (scale bar, 10 μm) for pSer‐10‐H3 (pSer‐10‐H3, green; DAPI—nuclear marker, blue) in cortical neurons (DIV 10) treated with *cis*‐RSV and *trans*‐RSV (50 μM) alone or in combination with *nMAβ*_*42*_ (50 nM) for 24 h. The graph represents pSer‐10‐H3 for *n* = 30 neurons per condition for *N* = 3 independent experiments with *p* values represented in the figure (* ≤ 0.05, ** ≤ 0.01, *** ≤ 0.001, and **** ≤ 0.0001). Statistical analysis was done using 2 way ANOVA with Tukey's multiple comparisons test.

To determine if *cis*‐RSV would counteract the effects of nMAβ_42_, we treated rat cortical neurons (DIV 9/10) with nMAβ_42_ and *cis*‐RSV and *trans*‐RSV alone and in combination. We found that *cis*‐RSV rescued nMAβ_42_‐induced TyrRS depletion, whereas *trans*‐RSV exacerbated it (Figure 7A). Furthermore, we found that *cis*‐RSV protected the cortical neurons against nMAβ_42_‐induced neurite degeneration, and in contrast, *trans*‐RSV exacerbated it (Figure 7B). Consistent with the above results, *cis*‐RSV protected cortical neurons against nMAβ_42_‐induced accumulation of DNA damage, whereas *trans*‐RSV alone induced it and exacerbated nMAβ_42_‐induced DNA damage accumulation (Figure 7C). Since histone H3 phosphorylation on the serine 10 residue (p‐Ser‐H3) is increased in AD brains [112], to further define the extent to which our in vitro system can mimic the cellular phenotypes identified in AD brains, we measured changes in p‐Ser‐H3 levels. We found that *cis*‐RSV rescued nMAβ_42_‐induced accumulation of p‐Ser‐H3, whereas *trans*‐RSV alone induced it and exacerbated Aβ_42_nM‐induced accumulation of p‐Ser‐H3 (Figure 7D). Together, these results suggest that *cis*‐RSV‐mediated depletion of tau (Figure 6D) might contribute to its ability to protect against nMAβ_42_‐mediated neurotoxic effects, and in contrast, *trans*‐RSV‐mediated accumulation of tau (Figure 6D) and translation inhibition [11] might contribute to *trans*‐RSV‐mediated recapitulation of nMAβ_42_‐mediated neurotoxic effects in primary neurons.

### 2.8 *Cis*‐RSV Protects Against Tyrosine‐Mediated Transcription Inhibition and Facilitates TOP1 and TOP2β‐Mediated Transcription Activation in Neurons

**FIGURE 8:**
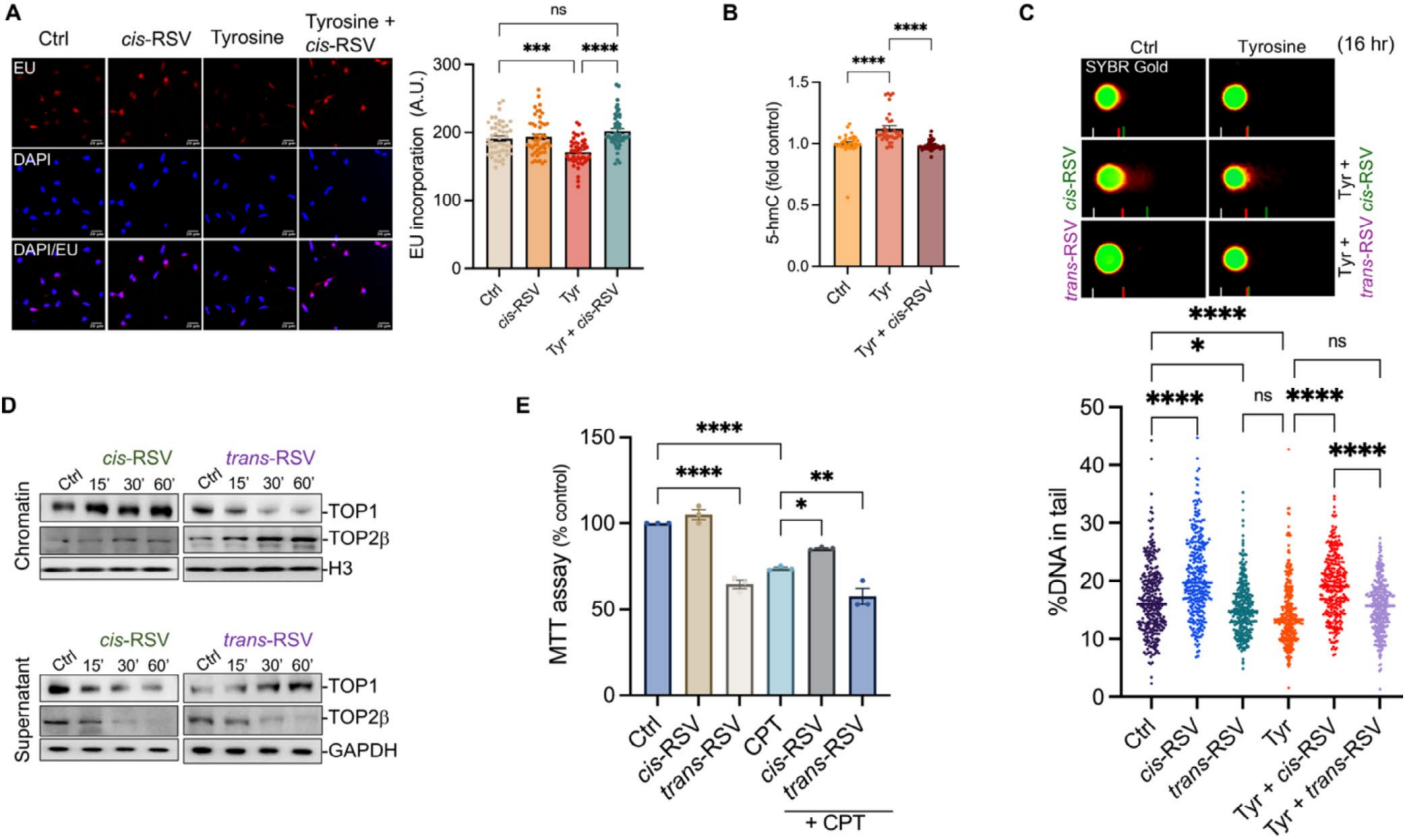
*cis*‐Resveratrol protects against tyrosine‐mediated transcription inhibition and CPT‐induced neurotoxicity. (A) *cis‐*RSV restores EU incorporation in tyrosine‐treated neurons. Quantitative IF analysis of EU incorporation in nascent RNA in primary cortical neurons treated with tyrosine (500 μM) or *cis*‐RSV (50 μM) alone or in combination for 4 h followed by the addition of EU for 30 min (*n* = 40 neurons per condition for *N* = 3 independent experiments). Statistical analysis was done using 2‐way ANOVA with Tukey's multiple comparisons test. (B) *cis*‐RSV prevents tyrosine‐induced 5‐hmC accumulation in neurons. Quantitative IF analysis of 5‐hmC in rat cortical neurons treated with tyrosine (500 μM) alone or in combination with *cis*‐RSV (50 μM) for 4 h (*n* = 30 neurons per condition for *N* = 3 independent experiments). Statistical analysis was done using 2 way ANOVA with Tukey's multiple comparisons test. (C) *cis*‐RSV and *trans*‐RSV exhibit differential effects on tyrosine‐mediated DNA repair. Quantification of DNA in comet tails from primary cortical neurons (DIV 9/10) treated with *cis*‐RSV and *trans*‐RSV (50 μM) alone or in combination with tyrosine (1 mM) for 16 h (*n* = 300 nuclei per condition for *N* = 3 independent experiments). Statistical analysis was done using 2‐way ANOVA with Tukey's multiple comparisons test. (D) *cis‐RSV* and *trans‐*RSV exhibit opposite effects on TOP1 recruitment to the chromatin. Primary cortical neurons were treated with *cis*‐RSV and *trans*‐RSV (50 μM) for up to 1 h, followed by WB analysis of chromatin‐bound TOP1 and TOP2β using their corresponding antibodies. (E) *Cis*‐RSV and *trans*‐RSV have opposing effects on CPT‐induced neurotoxicity. Neuronal survival was quantified using MTT assay after primary cortical neurons were pre‐treated with *cis*‐RSV and *trans*‐RSV (50 μM) for 24 h and CPT (10 μM) for 24 h, *n* = 3. Statistical analysis was done using 2‐way ANOVA with Šídák's multiple comparisons test. Data are presented as mean ± SEM from three independent experiments and *p* values are indicated in the figures (* ≤ 0.05, ** ≤ 0.01, *** ≤ 0.001, and *** ≤ 0.0001).

Since tyrosine inhibited transcription and activated neuronal DNA repair (Figure 1A,C), and we previously demonstrated that *cis*‐RSV protects against tyrosine‐mediated oxidative stress [11], we hypothesized that treatment with *cis*‐RSV would protect against tyrosine‐mediated transcription inhibition and associated activation of neuronal DNA repair. To test this, we treated rat cortical neurons (DIV 9/10) with tyrosine alone or in combination with *cis*‐RSV for 4 h followed by a 30 min pulse labeling using EU. Quantification of EU incorporation into nascent RNA showed that *cis*‐RSV protects against tyrosine‐mediated transcription inhibition (Figure 8A). Additionally, we treated rat cortical neurons (DIV 9/10) with tyrosine alone or in combination with *cis*‐RSV to assess its effects on tyrosine‐mediated 5‐hmC induction (Figure 3B). We found that *cis*‐RSV protects against tyrosine‐mediated accumulation of 5‐hmC (Figure 8B), providing a potential molecular basis for *cis*‐RSV‐mediated protection against tyrosine‐induced transcription inhibition.

To evaluate the effect of *cis*‐RSV on tyrosine‐mediated activation of DNA repair, we treated rat cortical neurons (DIV 9/10) with tyrosine alone, *cis*‐RSV and *trans*‐RSV, or their combinations for 16 h. Intriguingly, neurons treated with *trans*‐RSV showed decreased DNA fragments in the comets and did not rescue tyrosine‐mediated inhibition of DNA fragments in the comets (Figure 8C). In contrast, *cis*‐RSV treated neurons showed increased amounts of DNA fragments in the comets alone and in combination with tyrosine (Figure 8C), indicating a novel mechanism of *cis*‐RSV‐mediated protection against tyrosine‐induced neurodegeneration [11]. Given the differential effects of tyrosine and phenylalanine on TOP1 and TOP2β recruitment to chromatin (Figure 2A,B) and the fact that *trans*‐RSV induces TOP2‐induced DSBs [113], we investigated whether *cis*‐RSV and *trans*‐RSV would differentially regulate TOP1 and TOP2β recruitment to chromatin. To determine this, we treated rat cortical neurons (DIV 9/10) with *cis*‐RSV and *trans*‐RSV for up to 1 h and assessed the association of TOP1 and TOP2β in the chromatin fraction. *Cis*‐RSV facilitated the recruitment of TOP1 along with TOP2β to chromatin, whereas *trans*‐RSV decreased TOP1 levels while increasing TOP2β recruitment to the chromatin (Figure 8D), suggesting that *cis*‐RSV and *trans*‐RSV may trigger distinct transcriptional outcomes in primary neurons. Building on our previous finding that *cis*‐RSV protects against tyrosine and ETO‐induced neurotoxicity [11], we hypothesized that *cis*‐RSV would protect against CPT (TOP1 inhibitor)‐mediated neurotoxicity. We treated rat cortical neurons with CPT alone or in combination with *cis*‐RSV and *trans*‐RSV for 24 h and measured neuronal viability using a MTT assay. *Cis*‐RSV protected against CPT, while *tran*s‐RSV exacerbated its neurotoxicity (Figure 8E), further supporting our conclusion that *cis*‐RSV rescues tyrosine‐mediated transcription inhibition (Figure 8A), potentially by facilitating the resolution of both TOP1 and TOP2β‐induced DNA damage in primary neurons.

### 2.9 *Cis*‐RSV Downregulates Gene Expression Associated With Multiple Neurodegenerative Diseases and Viral Infections in Human Embryonic Stem Cell (hESC)‐Derived Neurons

**FIGURE 9:**
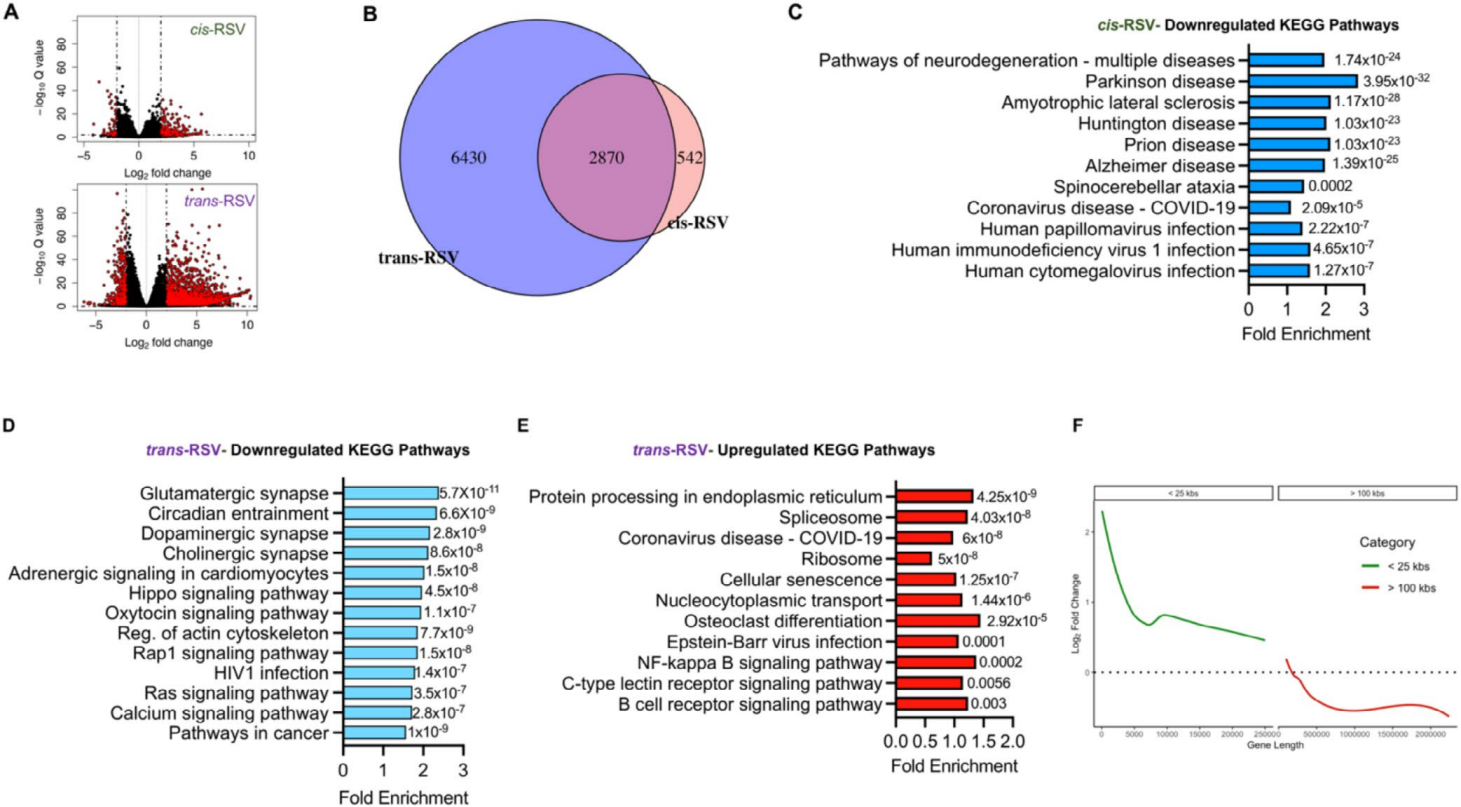
*cis*‐Resveratrol induces broad‐spectrum neuroprotective and anti‐viral response gene expression in hESC‐derived primary neurons. (A) *trans*‐RSV treatment results in a higher number of differentially expressed genes compared to *cis*‐RSV. Volcano plots show log2 fold changes (*x*‐axis) and ‐log 10 adjusted *p* values (*y*‐axis) for *cis*‐RSV (top) and *trans*‐RSV (bottom). Red dots indicate significantly differentially expressed genes. (B) Venn diagram illustrating the overlap of differentially expressed genes between *cis*‐ and *trans*‐RSV treatments. (C) Kyoto Encyclopedia of Genes and Genomes (KEGG) pathway enrichment analysis of genes downregulated by cis‐RSV. (D and E) Downregulated (D) and upregulated (E) KEGG pathways of trans‐RSV‐regulated mRNAs. (F) *trans*‐RSV increases expression of short genes and inhibits the expression of long genes. Line plot showing log2 fold changes in expression for short genes(< 25 kbs, green) and long genes (> 100 kbs, red) following *trans*‐RSV treatment. LOESS regression was applied to smooth the data across gene length categories.

DSB accumulation in AD‐affected neurons activates gene expression profiles with significant enrichment of genes implicated in inflammatory responses, accompanied by a reduction in genes associated with synaptic processes and neuronal identity, common in both sporadic and familial AD [ 44, 114]. ETO treatment recapitulates DSB‐induced neurodegenerative disease signatures in primary neuron cultures [44]. Similarly, coronavirus disease 2019 (COVID‐19) increases plasma phenylalanine levels [115, 116, 117] and the transcriptomic signature of severely affected COVID‐19 brains mirrors those seen in old age [118]. Our re‐analysis of the published data [119] showed that phenylalanine decreased the expression of long genes (Figure S2A), suggesting that increased phenylalanine levels in COVID‐19 patients [115, 116, 117] might have contributed to the aging‐like transcriptomic signature by inhibiting the expression of long genes [28, 29] in severely affected COVID‐19 brains [118]. Our previous work [11] demonstrated that *cis*‐RSV and *trans*‐RSV have differential effects on ETO‐induced neurotoxicity in rat cortical cultures, with only *cis*‐RSV providing protection. Since *trans*‐RSV recruited TOP2β with concomitant removal of TOP1 from the chromatin (Figure 8D) which mimicked the effect of phenylalanine (Figure 2B), we hypothesized that *cis*‐RSV and *trans*‐RSV would differentially affect neuronal gene expression, especially long genes and genes implicated in inflammatory pathways. We generated hESC‐derived neurons using a dual‐SMAD inhibition strategy that leads to the development of neurons from primarily the cortical excitatory lineage [120, 121]. Neurons were grown for 54 days, and we confirmed that *cis*‐RSV and *trans*‐RSV have opposing effects on TyrRS levels in hESC‐derived neurons (Figure S2B). For RNA‐Seq, hESC‐derived neurons were treated with *cis*‐RSV and *trans*‐RSV for 24 h. RNA‐Seq analysis showed that *cis*‐RSV treatment modulated a smaller subset of gene expression changes compared to *trans*‐RSV (Figure 9A,B) and *cis*‐RSV downregulated pathways associated with multiple neurodegenerative diseases and viral infections, including AD and COVID‐19, suggesting that *cis*‐RSV is a broad‐spectrum neuroprotective and anti‐viral compound (Figure 9C). Major pathways that increased with *cis*‐RSV include O‐glycan biosynthesis and fatty acid degradation (Figure S2C).

Interestingly, *trans*‐RSV downregulated pathways that included synapse‐associated functions, regulation of actin cytoskeleton, and circadian entrainment (Figure 9D). In contrast, *trans*‐RSV upregulated pathways that included NF‐κB‐dependent inflammatory gene expression along with pathways associated with viral infections, including COVID‐19, ribosome biogenesis, and cellular senescence (Figure 9E). Further analysis showed that *trans*‐RSV decreased the expression of long genes (> 100 kbs) while increasing the expression of short genes (Figure 9F), supporting our hypothesis that *trans*‐RSV exhibits differential effects on neuronal gene expression, particularly long genes and genes associated with inflammatory pathways potentially by activating TOP2β‐induced DSBs [113]. Furthermore, our analysis showed that the common differentially regulated gene profile by phenylalanine and *trans*‐RSV treatment includes an AD‐like gene signature (Figure S2D), suggesting that phenylalanine accumulation in AD brains [11] and COVID‐19 [115, 116, 117] may contribute to AD progression and aging. Despite differential effects on TyrRS levels (Figure S2B), both *cis*‐ and *trans*‐RSV significantly increased the mRNA levels of TyrRS in hESC‐derived neurons (1.5 and 2.8‐fold, respectively, data not shown), supporting the report that AD brains exhibit strong proteomic disease‐related changes not observed at the RNA level [75]. Together, these results suggest that *cis*‐RSV stimulates tubulin tyrosination, protects against TOP1/TOP2β‐mediated neuronal DNA damage accumulation, and facilitates TOP1/TOP2β‐mediated transcriptional activation while inhibiting the expression of inflammatory response genes associated with viral infections.

## 3 Discussion

Emerging research suggests that DNA repair factors are overexpressed in AD brains [122, 123], which prevent the induction of adaptive DNA breaks at the promoters of nervous system genes [123, 124], leading to the downregulation of gene expression pathways associated with cognition and memory formation [30, 31]. Further supporting these observations, a recent work showed that overactivation of faithful DNA repair causes aging [125]. Although transcription is inhibited during aging [27, 28], and overexpressed DNA repair factors may mitigate the induction of adaptive DNA breaks at the promoters of nervous system genes in AD brains [122, 123, 124] and may contribute to aging [125], physiological factors that activate age‐dependent DNA repair and transcription inhibition remain unknown. Here we demonstrate that tyrosine and phenylalanine, which are increased during aging [11, 63, 64] differentially regulate TOP1 and TOP2β‐induced DNA breaks, 5‐hmC/5‐mC levels, and global transcription and DNA repair in primary neurons. Tyrosine inhibits both PARP1‐dependent poly‐ADP‐ribos(PAR)ylation [126] and transcription and protects against ETO‐induced DSBs, which are repaired through non‐homologous end‐joining (NHEJ) [127]. PARP1 is involved in transcription activation [128] but histone serine‐ADP‐ribosylation facilitates PARP1 removal from damaged DNA [129] and inhibits transcription [71], suggesting that tyrosine‐mediated H3‐ADP‐ribosylation might also contribute to transcription inhibition. However, whether tyrosine‐mediated activation of NHEJ contributes to aging [28, 29] and ad [ 30, 31]‐associated downregulation of long genes, potentially by activating DNA repair at sites of adaptive DNA breaks at the promoters of long genes, requires further exploration. In contrast, phenylalanine increased γ‐H2AX levels along with increased global transcription and protection against CPT. These observations suggest that PARP1‐mediated inhibition of Ku‐dependent DNA repair [130] along with PARylation of TOP1 [131] may facilitate phenylalanine‐induced transcription to protect against CPT‐induced DNA damage [132]. Notably, phenylalanine depleted chromatin‐associated TyrRS along with TOP1 while activating SAR and protecting against CPT‐induced neurotoxicity, which is repaired through homologous recombination (HR) [133] or alternative end‐joining (alt‐EJ) pathway [134] regulated by PARP1 [135, 136]. Since terminally differentiated neurons are defective in HR [11] and sensitive to PARP inhibitors [11], and alt‐EJ is active in aging cells [137], whether tyrosine and phenylalanine activate alt‐EJ to protect against ETO/TOP2β‐ and CPT/TOP1‐induced DSBs and SSBs, respectively, during aging needs to be explored in the future. Wakefulness and explorative activities that stimulate cognition and memory increase DNA damage in neurons [79, 80]. In this context, it is interesting to note that caffeine, which decreases MRE11 [138] and DNA repair [139, 140, 141] protects against aging [142] and ad [ 143], and improves cognitive performance [144, 145]. Together, these observations highlight the significance of the development of novel inhibitors of tyrosine and phenylalanine‐induced neuronal DNA repair as potential therapeutics against aging and age‐associated neurodegenerative diseases such as AD.

*cis*‐RSV retained both TOP1 and TOP2β along with TyrRS on the chromatin while inhibiting SAR [11] and protected against CPT and tyrosine‐induced transcription inhibition. These findings indicate an essential role of nuclear TyrRS in facilitating TOP1 and TOP2β‐mediated long gene expression by inhibiting tyrosine and phenylalanine‐mediated DNA repair. Our finding that phenylalanine induces TOP2β‐mediated DSBs is consistent with the reports that transcription is overactivated in failing hearts [146, 147], TOP2β‐induced DSBs are responsible for cardiotoxicity induced by DNA damaging agents [148], and elevated serum phenylalanine triggers heart failure [65] and cardiac aging [66] and its supplementation decreases lifespan in *Caenorhabditis elegans* [149]. In contrast, downregulation of the tyrosine degradation pathway extends lifespan [150] and governs the response to protein restriction [151] in Drosophila. Since we found that tyrosine inhibited global transcription, and slowing down transcription elongation in worms and flies extended their lifespan [28], whether age‐dependent increase in serum tyrosine [11] is part of a protective stress response against phenylalanine‐induced toxic effects needs to be explored in the future. CPT inhibits the transcription of ribosomal RNA [41, 152]. However, whether tyrosine‐mediated recruitment of TOP1 and TyrRS to chromatin is involved in tyrosine‐induced DNA damage in 1 h and tyrosine‐mediated inhibition of translation [52] also remains to be established in the future.

**FIGURE 10:**
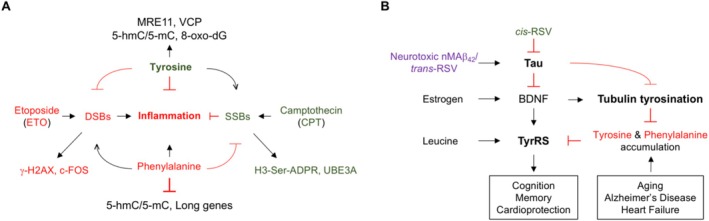
Illustration of the mechanism of action of tyrosine and phenylalanine‐mediated regulation of inflammation and tau/Aβ‐mediated regulation of neuronal TyrRS. (A) Tyrosine and phenylalanine have opposing effects on DSB‐induced inflammation. Tyrosine increases 5‐hmC/5‐mC and 8‐oxo‐dG and mimics the action of CPT in facilitating the recruitment of TOP1 to chromatin and increasing UBE3A. TOP1‐induced SSBs are potent inhibitors of inflammatory gene expression. The tyrosine‐mediated increase in histone serine‐ADPR, MRE11, and VCP activates DSB repair to protect against ETO. Phenylalanine mimics the action of ETO in facilitating the recruitment of TOP2β to the chromatin and facilitates the expression of inflammatory genes. While tyrosine‐mediated DSB repair might contribute to its anti‐inflammatory effects, in contrast, phenylalanine‐mediated SSB repair might alleviate the anti‐inflammatory effects of TOP1‐induced SSBs and the transcription of long genes. (B) *tau* and neurotoxic nMAβ_42_ are negative regulators of neuronal TyrRS, and cis‐RSV decreases tau and protects against the neurotoxic effects of nMAβ_42_. Tau and neurotoxic nMAβ_42_ negatively regulate neuronal TyrRS, potentially by inhibiting tubulin tyrosination and BDNF. Estrogen increases the expression of BDNF, which is a potent stimulator of neuronal tubulin tyrosination. Leucine increases neuronal TyrRS, which correlates with cognition and memory and protects against heart failure. Aging, AD, and heart failure increase serum tyrosine and phenylalanine levels, suggesting that *cis*‐RSV‐mediated depletion of tau and activation of tubulin tyrosination might help to mitigate age‐associated health decline.

5‐hmC is preferentially enriched in tissue‐specific gene bodies of highly expressed genes and at active enhancers [153], limiting the inflammatory response [48]. Therefore, 5‐hmC is a crucial mechanism in the epigenetic modulation of cell‐type specific transcription required for maintaining cell identity, and its levels are highest in the neurons [154, 155] and increase during aging [156, 157]. However, 5‐hmC is enriched in intragenic regions in AD brains [158] and TET1 overexpression impairs hippocampus‐dependent long‐term memory [159] and TET2 loss shows a neuroprotective effect in Parkinson's disease (PD) [160], indicating that tight regulation of 5‐hmC is required for normal neuronal functions and survival. Interestingly, PARP1‐dependent PARylation inhibits TET‐dependent 5‐hmC formation [161]. Since tyrosine and phenylalanine have differential effects on 5‐hmC levels and protection against CPT, these observations suggest that tyrosine and phenylalanine may have differential effects on PARP1‐dependent PARylation of TOP1 and TET. Recent works show that single‐stranded DNA (ssDNA) generated in response to DNA damage activates innate immune signaling [162] whereas 5‐hmC is an inhibitor of innate immune signaling [48] and inflammation‐associated transcriptional activation decreases 5 hmC [163]. CPT increases 5‐hmC [45] and exhibits anti‐inflammatory effects [43], and in contrast, ETO shows pro‐inflammatory effects [44]. Therefore, our finding that phenylalanine‐treated neuronal cultures sustain DNA fragments and exhibit decreased 5‐hmC, and tyrosine‐treated cultures exhibit decreased DNA fragments and increased 5‐hmC align with the reported pro‐inflammatory effects of phenylalanine [119] and anti‐inflammatory effects of tyrosine [164] as depicted in Figure 10A. However, since loss of 5‐hmC triggers tumorigenesis and chemoresistance in cancer cells [165], whether the novel function of tyrosine in transcription inhibition and 5‐hmC induction contributes to age‐associated transcription inhibition [27, 28], and increased incorporation of tyrosine in proteins upregulated in drug resistant cancer cells [10] remains to be explored.

Here, we also found that tau acts as an endogenous negative regulator, while estrogen and leucine act as positive regulators of neuronal TyrRS, potentially through their regulatory effects on BDNF and protein synthesis in cortical neurons. Estrogen enhances cognition and memory formation in females [166] and young women exhibit significantly lower serum tyrosine levels compared to male counterparts [11]. Since cortical TyrRS was higher in young/adult female mice and tau depletion increased TyrRS only in male mice, our study provides novel insights into a potential molecular basis for the higher resilience of women to the detrimental effects of tau [59, 60] and better memory and processing speed in women than men in middle age [58] potentially mediated through estrogen‐dependent induction of TyrRS and USP11 [95] leading to activation of global protein synthesis [61, 62]. Notably, while tau facilitates TIP5‐mediated transcriptional repression [167] potentially mediated through PARP1‐dependent formation of silent chromatin [168], estrogen induces topoisomerase‐mediated DNA breaks [34, 169] to facilitate PARP1‐dependent transcription [34, 170]. In contrast, postmenopausal women exhibit a significant increase in serum tyrosine levels [11] and tau accumulation [171, 172] compared to their male counterparts, and they are also at increased risk of developing AD [ 101, 102] and cardiovascular diseases (CVDs) [173]. Elevated tyrosine promotes heart failure and neurodegeneration by depleting nuclear TyrRS in cardiomyocytes [17] and neurons [11], respectively. Similarly, tyrosine and phenylalanine inhibit tubulin tyrosination in cortical neurons, and tau triggers tubulin detyrosination in failing hearts [20] and neurons [19]. Therefore, tau accumulation in post‐menopausal women may exacerbate tyrosine/phenylalanine‐mediated nuclear TyrRS depletion [11] and inhibition of tubulin tyrosination in the heart and brain. Interestingly, while BDNF, which increases tubulin tyrosination [92], H3‐Ser‐ADPR and TyrRS in neurons [11] protects against heart failure [174], *trans*‐RSV, which decreases tubulin tyrosination, H3‐Ser‐ADPR and TyrRS, increases the risk of CVDs [175]. Therefore, the inhibitory effect of tau on BDNF [93] and TyrRS, which activates H3‐Ser‐ADP‐ribosylation [11], is consistent with our previous finding that H3‐Ser‐ADP‐ribosylation is decreased in AD brains [11], and patients treated with tamoxifen (that increases H3‐Ser‐ADPR [71]) are protected against AD [ 176, 177] and CVDs [178, 179]. These observations might thus provide a potential molecular basis for HT‐related risk of dementia [180] and CVDs [173], increased CVD risk [175], and brain volume loss in AD patients [181] treated with high‐dose *trans*‐RSV. Estrogen, being a regulator of microtubule function as well [182], it would be interesting to determine how depletion of estrogen in aged mice shifts TyrRS expression, tubulin tyrosination, protein synthesis, and tau/TIP5‐mediated transcription inhibition, which may tip the balance toward a decrease, leading to cognitive decline. Since tyrosine is also an inhibitor of protein synthesis and global transcription, it is highly possible that the absence of female sex‐specific molecular pathways, especially estrogen‐mediated pathways, may exacerbate tau and tyrosine‐mediated TyrRS depletion [11], inhibition of tubulin tyrosination, and transcription inhibition, contributing to increased risk of AD [ 101, 102] and CVDs [173] in post‐menopausal women.

Inflammation and reduced fatty acid metabolism are hallmarks of aging [183]. Therefore, factors that mitigate inflammation along with the activation of fatty acid degradation are considered mediators of the health benefits of geroprotective interventions [184]. In this context, our finding that *cis*‐RSV downregulates gene expression associated with multiple viral infections including COVID‐19 while increasing gene expression associated with increased fatty acid degradation indicates the potential of *cis*‐RSV to mitigate age and COVID‐19‐related health decline. Tyrosinated α‐tubulin at the neuronal injury site not only activates pro‐regenerative transcriptional programs important for neuronal regeneration [185] but also regulates synaptic function [22] and memory formation [186]. Since tau increases tubulin detyrosination in the brain [19] and heart [20] as well as AD brains [22] and failing hearts exhibit accumulation of detyrosinated tubulin [20, 187, 188], it is highly likely that *cis*‐resveratrol‐mediated activation of tubulin tyrosination and depletion of tau, along with protection against tyrosine‐mediated inhibition of transcription and accumulation of 5‐hmC/5‐mC, might have contributed to the reported cognitive benefits of low‐dose resveratrol (that exists as *cis*‐RSV [126, 189]) in post‐menopausal women [190] and cardioprotective effects in heart failure patients [191] as depicted in Figure 10B.

Together, these results suggest that *cis*‐resveratrol might restore protein synthesis and transcription in aging tissues/organs along with increased tubulin tyrosination and facilitated tau depletion. Importantly, even a ~30% decrease in CNS tau in the adult brain is sufficient to alleviate Aβ‐induced neuronal abnormalities in the brain [26]. Therefore, our finding that tyrosine/phenylalanine and leucine have opposite effects on neuronal TyrRS along with previous reports that higher phenylalanine is associated with increased all‐cause mortality [64] whereas higher leucine levels are associated with decreased all‐cause mortality [64], protection against CVDs [53], skeletal muscle dysfunction [54] and cognitive dysfunction in humans [55, 56], suggest that a supplement combination of leucine with *cis*‐resveratrol that mimics a ‘tyrosine‐free’ conformation in TyrRS [189] may provide beneficial effects against various age‐associated neurocognitive, degenerative and metabolic disorders including CVDs.

## 4 Experimental Procedures

### 4.1 Animals

WT and *Tau* KO mice were obtained from The Jackson Laboratories (stock #007251) and housed in a controlled environment at Georgia State University (GSU), with strict adherence to a 12‐h light/12‐h dark cycle and proper food and water access at all times. Validation of genotype was performed as previously described [84]. Sprague Dawley rats were purchased from the Charles River Laboratories, Crl:CD(SD) and housed in a controlled environment at the University of South Carolina (USC), with strict adherence to a 12‐h light/12‐h dark cycle and proper food and water access at all times. Animal care and use were carried out following the National Institutes of Health Guidelines for the Use of Animals, using approved protocols by the GSU and USC Institutional Animal Care and Use Committees (IACUC).

### 4.2 Primary Neuronal Culture

Primary cortical neurons were harvested from 18‐day‐old Sprague Dawley rat pups using Hibernate E (BrainBits) and dissociated with the Neural Tissue Dissociation kit (Miltenyi Biotec) as we described recently [11]. Briefly, the cortices were minced and incubated in a pre‐heated enzyme mix at 37°C for 15 min. The tissues were then strained using a 40 μm cell strainer, washed, and centrifuged. The neurons were then cultured on tissue culture plates coated with 50 μg/mL poly‐D‐Lysine (Sigma Aldrich). The culture medium consisted of NBActive‐1 medium (BrainBits) supplemented with 100 U/mL of Penicillin–Streptomycin (Life Technologies), 2 mM L‐Glutamine (Life Technologies), and 1× N21 supplement (R&D Systems).

### 4.3 Immunoblotting of Murine Cortical Lysates

Dissected cortices from 2 to 3 month‐old WT and *Tau* KO mice or 4 month‐old and 12 month‐old WT mice of both sexes were homogenized in 10 volumes of HEPES‐buffered sucrose (0.32 M sucrose, 4 mM HEPES, pH 7.4) with 1 mM DTT and protease inhibitors (0.1 mM PMSF, 1 μM leupeptin, 0.15 μM aprotinin). Tissue homogenate was spun at 1000×*g* for 15 min at 4°C to remove the nuclear fraction. The resulting supernatant was used for immunoblotting procedures. Quantification of protein lysates was conducted using the Pierce BCA Protein Assay kit (Thermo Scientific, 23227). To prepare samples for loading, the protein samples were diluted with the extraction buffer mixed with complete NuPAGE LDS Sample Buffer (4×) (Thermo Scientific, NP007) to a final concentration of 1 μg/μL and heated for 15 min at 95°C in a heat block. Samples were loaded onto 10% SDS‐PAGE gels and run at 120 V. The proteins were then transferred to a 0.2 μm nitrocellulose membrane (Bio‐Rad, 1620112) at 100 V for 1 h at 4°C. Membranes were stained with the Revert 700 nm total protein stain for western normalization (LI‐COR Biosciences, 926–11011) and imaged on the LI‐COR Odyssey CLx scanner (low scan quality, 163 μm scan resolution, 700 nm channel, auto channel intensities) to confirm protein normalization across the samples. Post imaging, membranes were incubated for 1 h at room temperature under rocking conditions in 5% Blocking Grade Blocker solution (Bio‐Rad, 1706404) prepared in 1× Tris‐Buffered Saline (TBS). After blocking, membranes were incubated with the following primary antibodies diluted in 5% Blocking Grade Blocker solution (prepared as above) overnight at 4°C in a shaker: mouse monoclonal anti‐Tau‐1 antibody clone PC1C6 (1:1000; Millipore, MAB3420) and mouse polyclonal anti‐TyrRS antibody (1:500; Abcam, ab50961). After that, membranes were washed 3 times with TBS‐T (1× TBS with 0.1% Tween‐20) for 5 min each under rocking conditions and incubated with the following secondary antibodies for 1 h at room temperature in a rocker: IRDye 800CW goat anti‐mouse antibody (1:20,000; LI‐COR Biosciences, 926–32210) and IRDye 800CW goat anti‐rabbit antibody (1:15,000; LI‐COR Biosciences, 926–32211). Then, membranes were washed 2 times with TBS‐T and 1 time with TBS for 5 min each in a rocker and imaged on the LI‐COR Odyssey CLx scanner (low scan quality, 163 μm scan resolution, 700 nm and 800 nm channels, auto channel intensities). Post‐imaging, membranes were blocked again for 1 h at room temperature under rocking conditions in 5% Blocking Grade Blocker solution and incubated with the mouse monoclonal anti‐β actin antibody (1:3000; GeneTex, GT5512) overnight at 4°C in a shaker. After that, membranes were washed 3 times with TBS‐T for 5 min each in a rocker and incubated with the IRDye 800CW goat anti‐mouse antibody, as above. Then, membranes were washed 2 times with TBS‐T and 1 time with TBS for 5 min each under rocking conditions and imaged on the LI‐COR Odyssey CLx scanner (low scan quality, 163 μm scan resolution, 700 nm and 800 nm channels, auto channel intensities). Images were analyzed using the Image Studio Lite software (Li‐COR Biosciences) to quantify band intensities. Calculations and statistical analysis were performed in Microsoft Excel using the Student's *t*‐test (two‐tailed distribution, two‐sample unequal variance) for statistical significance for each protein after combining the data from each batch of mice, which were processed together.

### 4.4 Human Embryonic Stem Cell Culture

Stem cell cultures and neuronal differentiation were conducted as previously described [120, 121]. On day 21, 50,000 neurons were plated on 24 well plates coated with poly‐ornithine (Sigma) and laminin (20 μg/mL, Thermo‐Fisher). These cells were maintained by half‐media changes on alternate days using neural maintenance media (NMM). NMM contains 1:1 DMEM/F‐12 GlutaMAX (Life Technologies) and Neurobasal medium (Life Technologies) with 1× N2 supplement (Life Technologies), 1× B27 supplement (Life technologies Catalog), insulin (5 μg/mL, Sigma), L‐glutamine (1 mM, Life Technologies), sodium pyruvate (500 μM, Sigma), nonessential amino acids solution (100 μM, Life Technologies), β‐mercaptoethanol (100 μM, Sigma), penicillin–streptomycin (50 U/mL, Life Technologies). On day 54, the neurons were treated with *cis*‐RSV and *trans*‐RSV (50 μM each) for a period of 24 h, followed by the collection of cell pellets in ice‐cold PBS, using cell scrapers, to obtain cell pellets for RNA extraction.

### 4.5 RNA Extraction and Library Preparation

RNA and library preparation, and post‐processing of the raw data were performed by the USC CTT COBRE Functional Genomics Core. RNAs were extracted using Zymo Quick‐RNA MicroPrep Kit as per manufacturer recommendations, and RNA was cleaned by Zymo RNA Clean and Concentrator Kit (Zymo Research, Irvine, CA, USA). RNA quality was evaluated on an RNA‐1000 chip using Bioanalyzer (Agilent, Santa Clara, CA, USA). RNA libraries were prepared using an established protocol with NEBNExt Ultra II Directional Library Prep Kit (NEB, Lynn, MA). Each library was made with one of the TruSeq barcode index sequences and the Illumina sequencing done by Novogene (Sacramento, CA) with Illumina HiSeq PE150 (150 bp, pair‐ended). Sequences were aligned to the Human genome GRCh38.77 (GCA_000001405.15, ensemble release‐77) using STAR v2.4 [192]. Samtools (v1.2) was used to convert aligned sam files to bam files, and reads were counted using the featureCounts function of the Subreads package [193] using Homo_sapiens.GRCh38.77.gtf annotation file. Only reads mapped uniquely to the genome were used for gene expression analysis. Differential expression analysis was performed in R using the DeSeq2 package.

### 4.6 EU Incorporation Assay

Click‐iT RNA Alexa Fluor 594 Imaging Kit (Invitrogen, C10330) was used for the EU (5‐ethynyl Uridine) incorporation assay. Briefly, the stock solutions were prepared as per the manufacturer's instructions. After the treatment, a working solution of 100 mM stock of EU was added to the well for a final concentration of 50 μM in each well, followed by incubation for 30 min at 37°C. The media was then removed, and the wells were washed twice with PBS before fixing them using 4% paraformaldehyde for 15 min at room temperature. Cells were permeabilized and washed with PBS, followed with a 30‐min incubation with freshly prepared 1x Click‐iT solution and Click‐iT cocktail in the dark. After 30 min, cells were washed with Click‐iT rinse buffer. Primary antibody for MAP2 (Aves lab) was added, and cells were incubated overnight at 4°C, followed by secondary antibody incubation for 1 h at room temperature using Alexa Fluor 647 (anti‐chicken) from Invitrogen at a dilution of 1:1000. Finally, the coverslips were mounted using DAPI‐supplemented mounting medium, Prolong Gold Antifade (Invitrogen) and imaged with a Leica DMI6000 epifluorescent microscope using oil immersion 63x/NA 1.4 objective. The quantification for total EU levels in the nucleus was done using ImageJ (Version 1.53c), with matched imaging parameters for exposure, gain, and offset.

### 4.7 Immunoblotting for Primary Neuronal Cortical Cultures

Cultured primary rat cortical neurons (DIV 9/10) were prepared for analysis by washing with cold 1× PBS and lysing in cell lysis buffer. The lysates were then centrifuged at 15,000×*g* for 15 min at 4°C to separate the chromatin‐bound and soluble fractions, and equal amounts of protein were loaded onto a 4%–12% gradient gel (NuPAGE‐Invitrogen) for electrophoresis. The protein was transferred to a 0.2 μm NC membrane, and the membrane was blocked with 5% non‐fat milk in TBST. Primary antibodies were applied to the membrane and incubated overnight at 4°C, followed by incubation with secondary antibodies for 1 h at room temperature. The Immobilon ECL Ultra Western HRP Substrate was used to detect the proteins, and the luminescent image analyzer (ChemiDoc Imaging System, Bio‐Rad) was used for quantification. The western blots were quantified using ImageJ software (Version 1.53t).

### 4.8 List of Antibodies Used for Western Blotting

**Table:** 

Antibody	Company	Catalog No.	Dilution
α‐Tubulin	Proteintech	66,031‐1‐Ig	1:2000
Delta2‐tubulin	Millipore	AB3203	1:2000
DeTyr‐tubulin	Millipore	AB3201	1:1000
GAPDH	Cell Signaling Technology	2118	1:2000
H3	Proteintech	17,168‐1‐AP	1:1000
PARP1	Proteintech	66,520‐1‐Ig	1:1000
Poly (ADP‐Ribose) Polymer	Abcam	ab14459	1:1000
TOP1	Novus Biologicals	NBP1‐90365	1:500
TOP2β	Novus Biologicals	NBP1‐89527	1:1000
TTL	Proteintech	66,076‐1‐Ig	1:1000
Tyr‐tubulin	Millipore	**MAB1864‐I**	1:2000
TyrRS	Abcam	ab50961	1:1000
UBE3A	Cell Signaling Technology	7526	1:1000
c‐FOS	Cell Signaling Technology	2250	1:500
Phospho‐Histone H2A.X	Cell Signaling Technology	9718	1:1000
H3‐S10‐ADP‐Ribose	Bio‐RAD	HCA357	1:1000
MRE11	Cell Signaling Technology	4895	1:1000
VCP	Proteintech	10,736‐1‐AP	1:1000

### 4.9 Comet Assay

The cells were harvested using a cell scraper in chilled PBS and counted as reported previously [11]. The comet assay (Trevigen Inc., Gaithersburg, MD) was performed according to the manufacturer's protocol using alkaline conditions as we described recently [11]. Briefly, after electrophoresis, the slides were washed twice in deionized water for 5 min and immersed in 70% ethanol for 5 min. Subsequently, the slides were dried at 37°C for 30 min. DNA staining was done using SYBR Gold dye (Fisher Scientific, 1:10000 in Tris–EDTA buffer, pH 7.5) for 20 min in the dark at room temperature and then imaged using an epifluorescent microscope at 10× magnification. The images were quantified and scored for comet parameters such as tail length using the Tritek CometScore Freeware v1.5 image analysis software.

### 4.10 Immunofluorescence (IF)

Cultured cortical neurons at DIV 9–10 were fixed in 4% formaldehyde for 15 min, permeabilized, and blocked with 5% BSA (PBS) and 0.1% Tween20 for 30 min at room temperature. Primary antibodies were added and incubated overnight at 4°C, followed by secondary antibody incubation for 1 h at room temperature. Alexa Fluor 647 (anti‐chicken), Alexa Fluor 555 (anti‐mouse), and Alexa Fluor 488 (anti‐rabbit) from Invitrogen were used as secondary antibodies at a dilution of 1:1000. Coverslips were mounted with DAPI‐supplemented mounting medium, Prolong Gold Antifade (Invitrogen), and imaged with a Leica DMI6000 epifluorescent microscope using an oil immersion 63×/NA 1.4 objective. Total protein levels in neurons were quantified using ImageJ (Version 1.53c), with imaging parameters matched for exposure, gain, and offset. Neuronal γ‐H2AX foci were calculated as previously described [194].

### 4.11 List of Antibodies Used for IF

**Table:** 

Antibody	Company	Catalog No.	Dilution
MAP2	Aves labs	SKU: MAP	1:500
TyrRS	Novus Biologicals	NBP1‐32551	1:200
Tyr‐tubulin	Millipore	**MAB1864‐I**	1:1000
TTL	Proteintech	66,076‐1‐Ig	1:300
5mC	Cell Signaling Technology	28,692	1:500
5hmC	Cell Signaling Technology	51,160	1:500
p‐Tau	Cell Signaling Technology	20,194	1:800
Tau	Proteintech	66,499‐1‐Ig	1:800
8‐oxo‐2′‐dG	Abcam	ab48508	1:200
Phospho‐Histone H3 (Ser10)	Cell Signaling Technology	9701	1:400
Phospho‐Histone H2AX (Ser139)	Cell Signaling Technology	9178	1:400

### 4.12 Pharmacological Treatments

All drugs/inhibitors stock solutions (1000×) were prepared in DMSO or ethanol and diluted in culture media to their final concentration. The various compounds used for treatments and their stock concentrations are listed below.

**Table:** 

Compound	Catalog	Stock concentration	Final concentration	Solvent
L‐Tyr	194759, MP Biomedicals	100 mM	0.1–0.5 mM	PBS
L‐Phe	A13238, Alfa Aesar	100 mM	0.1–0.5 mM	PBS
L‐Leu	L8000, Sigma Aldrich	100 mM	0.1–0.5 mM	PBS
Etoposide	28435, Chem Implex	100 mM	10 μM	DMSO
Camptothecin	276721000, Acros Organics	100 mM	10 μM	DMSO
Parthenolide	0610, R&D Systems	10 mM	10 μM	DMSO
Paclitaxel	AC328420250, Acros Organics	10 μM	10 nM	DMSO
*cis*‐RSV	10004235, Cayman Chemicals	100 mM	50 μM	Ethanol
*trans*‐RSV	34092, Millipore‐Sigma	100 mM	50 μM	Ethanol
EpoY	SML2301, Sigma‐Aldrich	10 mM	10 μM	DMSO
β‐Estradiol	10006315, Cayman Chemical	1 mM	100 nM	Ethanol

### 4.13 Amyloid Beta Preparation

Aβ1‐42 was prepared as described previously [195]. In short, human amyloid β protein fragment 1–42 (Aβ_1–42_, A9810, Sigma Aldrich) stock solution of 100 μM was prepared for cell cultures in sterile H_2_O (0.01% DMSO). An additional incubation was performed for oligomer formation at 37°C for 48 h and confirmed the low molecular weight oligomer formation using western blots. The concentration used for treatment in neuronal culture was 50 nM unless specified otherwise.

### 4.14 DNA Fiber Analysis

Cortical neurons (DIV 9/10) were harvested in chilled PBS and processed for DNA fiber analysis as previously reported [11]. Briefly, cells were trypsinized, embedded in agarose plugs, and digested with proteinase K (0.5% SDS, 0.1 M EDTA, 1 mg/mL) at 50°C for 16 h. Plugs were dissolved in agarose (NEB, 50‐811‐726) for 16 h. Molecular combing was performed with the FiberComb System (Genomic Vision) using a 2 kb/μm stretching factor on vinylsilane coverslips (20 × 20 mm). After incubating at 60°C for 2 h, DNA was denatured (0.5 M NaOH +1 M NaCl) for 8 min. Coverslips were washed, dehydrated, and blocked with 3% BSA/1x PBS for 30 min. Primary antibodies α‐BrdU (BD #347,580, 1:100) and ssDNA (Millipore #MAB3034, 1:100) were incubated for 2 h at 37°C, followed by secondary antibodies α‐mouse AlexaFluor 594 and α‐rat AlexaFluor 488 (1:500) for 1 h. After washing, coverslips were dehydrated and mounted. DNA fibers were visualized using an EVOS FL fluorescence microscope (Thermo Scientific). ImageJ was used to count 300 fibers per condition for determining CldU incorporation ratios.

### 4.15 Cell Viability Assays

Rat cortical neurons (DIV 9/11) were seeded at a density of 20,000 cells/well in 96‐well plates. Cultured rat cortical neurons were incubated with MTT (0.5 mg/mL) for 2 h following treatment with the drugs for the indicated durations. Following the 2 h incubation, the insoluble formazan produced by reduction of MTT by NAD(P)H‐dependent mitochondrial oxidoreductases was solubilized using DMSO, under agitation and protected from light. The percentage of MTT reduced is measured by the difference in absorbance at 570 nm read in a spectrophotometer (Speactramax190, R Molecular Devices, UK). Results are presented as percent control (wells incubated with the vehicle).

### 4.16 Neurite Degeneration Index

Neurite degeneration index was calculated as described previously [24, 196]. Samples were imaged using ImageXpress Micro 4 at a magnification of 10x to capture the entire field of interest. The samples to be analyzed for neurite degeneration were stained using the standard immunofluorescence procedure with MAP2 (Alexa fluor 647, Invitrogen #A‐21449) for neurites and DAPI staining for the nucleus. Neurite degeneration was quantified using 5–6 regions of interest of equal sizes from each treatment condition. The analysis of neurite degeneration was done using ImageJ. The fluorescent images for MAP2 staining were binarized. Healthy intact neurites showed continuous tracts, while degenerated neurites appeared particulate due to fragmentation or beading. The particle analyzer module of ImageJ was used to calculate the percentage area of small fragments (size = 3–10 μm [2]) relative to the intact neurites (size > 25 μm [2]) from the binary images. The degeneration index (DI) was calculated as the ratio of fragmented neurite area to intact neurite area.

### 4.17 Publicly Available Data Set Analysis

Transcriptomic, proteomic, and protein‐specific correlation data for various disease parameters such as MMSE score, Braak stage, CERAD, and Aβ plaque levels and the effects of phenylalanine treatment on long gene expression were collected from a publicly available data [75, 119].

### 4.18 Statistical Analysis

The data was analyzed for statistical significance and differences between groups were determined. Depending on the number of groups being compared, either one‐way ANOVA with multiple comparisons without correction or two‐way ANOVA with multiple comparisons without correction were used. When comparing two groups, either paired or unpaired *t*‐tests were used. The data analysis was performed using GraphPad statistical analysis software.

## Conflicts of Interest

M.S. holds equity in Functional Longevity Labs Inc. All other authors declare no conflicts of interest.

## Supporting information


**Figure S1.** **Amyloid beta at nanomolar concentrations negatively regulates neuronal TyrRS via protein synthesis inhibition**. **A**. *Brain UPS11 levels correlate positively with cognition and memory*. Graph indicating the correlation between UPS11 and cognitive performance/AD progression from re‐analyzed publicly available AD brain proteome data. B. *Nanomolar concentrations of amyloid beta 42 (nMAβ*_*42*_ *) peptide decrease neuronal TyrRS*. Representative immunoblots and quantification for TyrRS and PheRSβ after treatment with nMAβ_42_ (50 nM) for up to 16 h using their specific antibodies, *n* = 3. C. *nMAβ*_*42*_ *decreases neuronal TyrRS in a dose‐dependent manner*. Representative immunoblots and quantification for TyrRS after treatment with nMAβ_42_ (5–50 nM) for up to 24 h using an anti‐TyrRS antibody, *n* = 3. D. *The reverse peptide of Aβ*_*42*_ (42‐1) *does not affect neuronal TyrRS*. Representative immunoblots and quantification for TyrRS after treatment with either nMAβ_42‐1_ (50–100 nM) or nMAβ_1‐42_ (50 nM) for 24 h using an anti‐TyrRS antibody, *n* = 3. E. *nMAβ*_*42*_ *induces eEF2 phosphorylation*. Representative immunoblots and quantification for eEF2 and p‐eEF2 after treatment with nMAβ_42_ (50 nM) for up to 16 h using their specific antibodies, *n* = 3. Statistical analysis was done using 2‐way ANOVA with Tukey’s multiple comparisons test. Data are presented as mean ± SEM from three independent experiments and *p* values are indicated in the figures (* ≤ 0.05, ** ≤ 0.01, *** ≤ 0.001, **** ≤ 0.0001).
**Figure S2.** Common differentially expressed genes (DEGs) in phenylalanine and *trans*‐RSV treated hESC‐derived neurons evoke AD‐like gene expression signature. A. *Phenylalanine inhibits long gene expression in hESC‐derived neurons*. Line plot depicting log2 fold change in gene expression across different gene lengths using publicly available data. The x‐axis represents gene length, and the y‐axis represents the log2 fold change in expression. The blue line indicates the differential expression trend across varying gene lengths. B. *cis‐ and trans‐RSV have opposing effects on TyrRS in hESC‐derived neurons*. Human ESC‐derived cortical neurons were treated with *cis*‐ and *trans*‐RSV (50 μM) for 8 h and changes in TyrRS were quantified using IF (*n* = 40 neurons per condition for *N* = 3 independent experiments). Statistical analysis was done using 2way ANOVA with Tukey’s multiple comparisons test. (*p* values * ≤ 0.05, ** ≤ 0.01, *** ≤ 0.001, **** ≤ 0.0001) C. *cis‐RSV upregulates genes involved in fatty acid degradation*. KEGG pathway enrichment for genes upregulated after treatment with *cis*‐RSV. D. *Phenylalanine and trans‐RSV evoke common DEGs profile including AD‐like gene expression signature in KEGG pathway analysis*. Pathway enrichment for KEGG pathways enriched for 866 DEGs common between phenylalanine and *trans*‐RSV.

## Data Availability

All data needed to evaluate the conclusions in the article are present in the article and/or the Supporting Information. The raw sequencing data and processed files have been deposited in the Gene Expression Omnibus (GEO) database under accession number GSE227181. Additional data related to this article may be requested from the authors. Correspondence and requests for materials should be addressed to Mathew Sajish, Email: mathew2@cop.sc.edu.
