# Supplementary material for: Tyrosine and Phenylalanine Activate Neuronal DNA Repair but Exhibit Opposing Effects on Global Transcription and Adult Female Mice Are Resilient to TyrRS/YARS1 Depletion
Source: IUBMB Life. 2025 Jun 6;77(6):e70030. doi: 10.1002/iub.70030 (PMC12142579; doi:10.1002/iub.70030)
Supplement: Supplementary file 1 — Figure S1. Amyloid beta at nanomolar concentrations negatively regulates neuronal TyrRS via protein synthesis inhibition. A. Brain UPS11 levels correlate positively with cognition and memory. Graph indicating the correlation between UPS11 and cognitive performance/AD progression from re‐analyzed publicly available AD brain proteome data. B. Nanomolar concentrations of amyloid beta 42 (nMAβ 42 ) peptide decrease neuronal TyrRS. Representative immunoblots and quantification for TyrRS and PheRSβ after treatment with nMAβ42 (50 nM) for up to 16 h using their specific antibodies, n = 3. C. nMAβ 42 decreases neuronal TyrRS in a dose‐dependent manner. Representative immunoblots and quantification for TyrRS after treatment with nMAβ42 (5–50 nM) for up to 24 h using an anti‐TyrRS antibody, n = 3. D. The reverse peptide of Aβ 42 (42‐1) does not affect neuronal TyrRS. Representative immunoblots and quantification for TyrRS after treatment with either nMAβ42‐1 (50–100 nM) or nMAβ1‐42 (50 nM) for 24 h using an anti‐TyrRS antibody, n = 3. E. nMAβ 42 induces eEF2 phosphorylation. Representative immunoblots and quantification for eEF2 and p‐eEF2 after treatment with nMAβ42 (50 nM) for up to 16 h using their specific antibodies, n = 3. Statistical analysis was done using 2‐way ANOVA with Tukey’s multiple comparisons test. Data are presented as mean ± SEM from three independent experiments and p values are indicated in the figures (* ≤ 0.05, ** ≤ 0.01, *** ≤ 0.001, **** ≤ 0.0001). Figure S2. Common differentially expressed genes (DEGs) in phenylalanine and trans‐RSV treated hESC‐derived neurons evoke AD‐like gene expression signature. A. Phenylalanine inhibits long gene expression in hESC‐derived neurons. Line plot depicting log2 fold change in gene expression across different gene lengths using publicly available data. The x‐axis represents gene length, and the y‐axis represents the log2 fold change in expression. The blue line indicates the differential expression trend across vary [file IUB-77-0-s001.docx]

**SUPPELEMENTARY FIGURE 1**

**
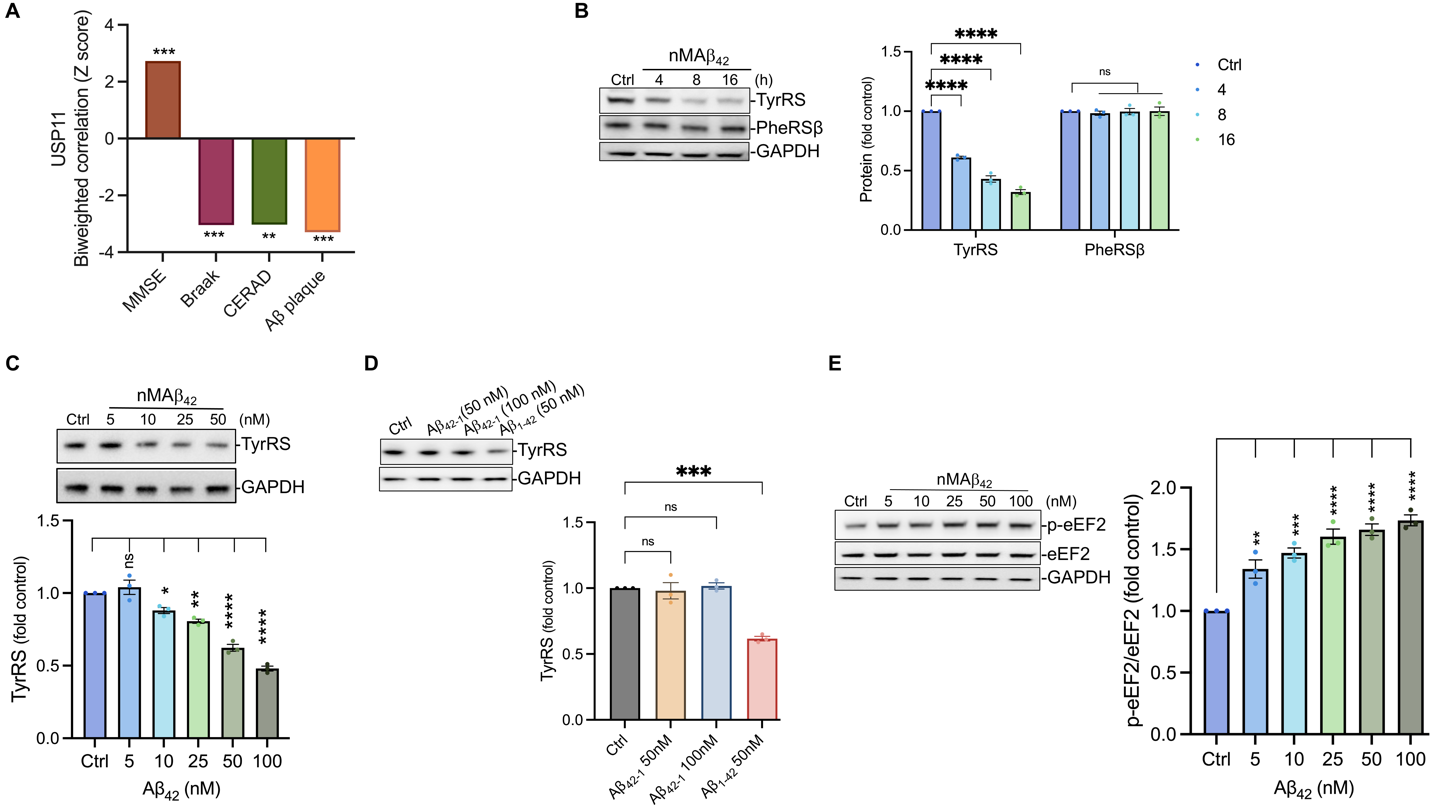
**

**Supplementary Figure 1**. **Amyloid beta at nanomolar concentrations negatively regulate neuronal TyrRS via protein synthesis inhibition**. **A.** *Brain UPS11 levels correlate positively with cognition and memory.* Graph indicating the correlation between UPS11 and cognitive performance/AD progression from re-analyzed publicly available AD brain proteome data. **B**. *Nanomolar concentrations of amyloid beta 42 (nMAβ_42_) peptide decrease neuronal TyrRS.* Representative immunoblots and quantification for TyrRS and PheRSβ after treatment with nMAβ_42_ (50 nM) for up to 16 hr using their specific antibodies, n=3. **C**. *nMAβ_42_ decreases neuronal TyrRS in a dose-dependent manner.* Representative immunoblots and quantification for TyrRS after treatment with nMAβ_42_ (5-50 nM) for up to 24 hr using an anti-TyrRS antibody, n=3. **D**. *The reverse peptide of Aβ_42_ (42-1) does not affect neuronal TyrRS.* Representative immunoblots and quantification for TyrRS after treatment with either nMAβ_42-1_ (50-100 nM) or nMAβ_1-42_ (50 nM) for 24 hr using an anti-TyrRS antibody, n=3. **E**. *nMAβ_42_ induces eEF2 phosphorylation.* Representative immunoblots and quantification for eEF2 and p-eEF2 after treatment with nMAβ_42_ (50 nM) for up to 16 hr using their specific antibodies, n=3. Statistical analysis was done using 2way ANOVA with Tukey’s multiple comparisons test. Data are presented as mean ± SEM from three independent experiments and *p* values are indicated in the figures (*=≤0.05, **=≤0.01, ***=≤0.001, ****=≤0.0001).

**SUPPELEMENTARY FIGURE 2**

**
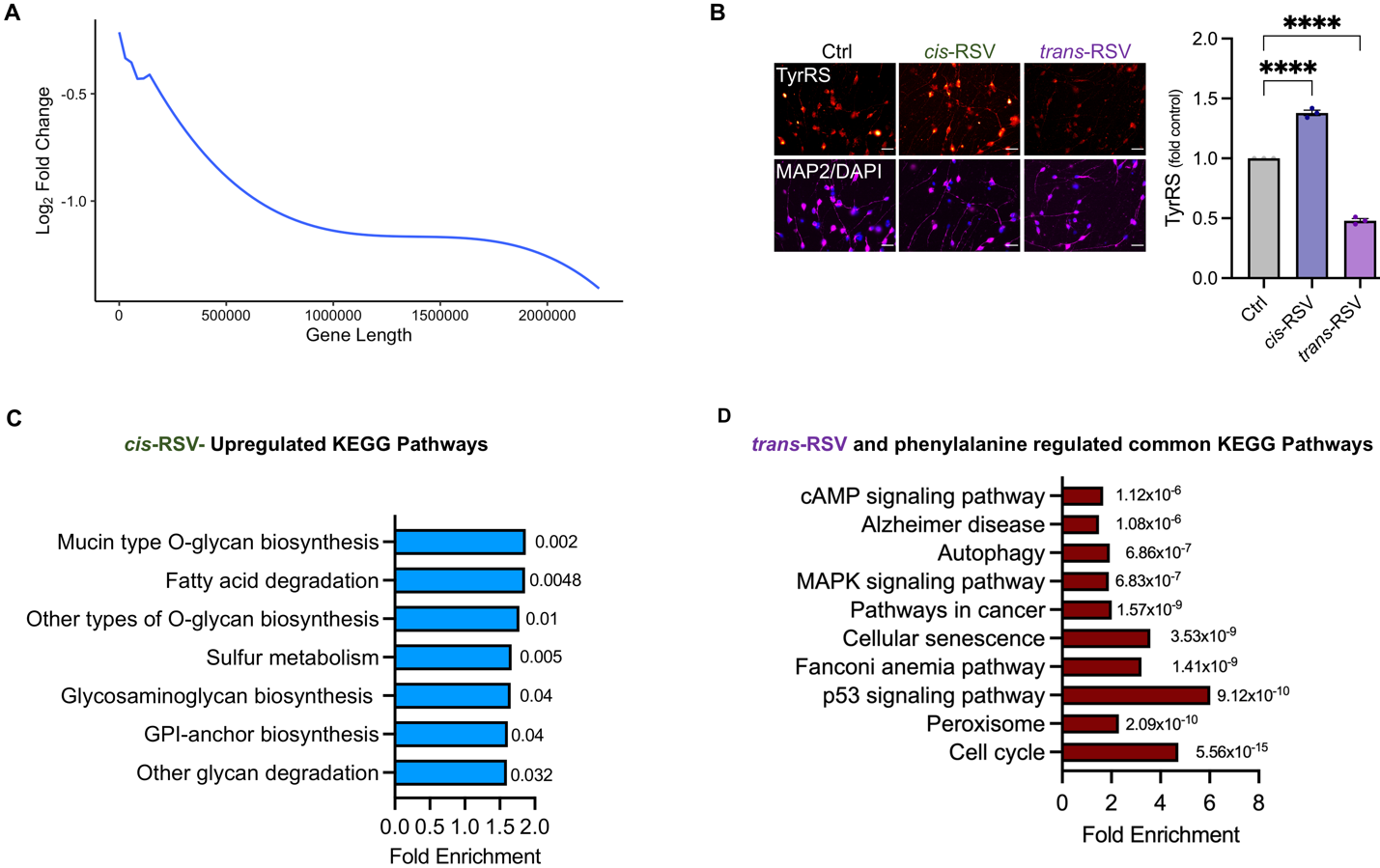
**

**Supplementary Figure 2. Common differentially expressed genes (DEGs) in phenylalanine and *trans*-RSV treated hESC-derived neurons evoke AD-like gene expression signature. A.** *Phenylalanine inhibits long gene expression in hESC-derived neurons.* Line plot depicting log2 fold change in gene expression across different gene lengths using publicly available data. The x-axis represents gene length, and the y-axis represents the log2 fold change in expression. The blue line indicates the differential expression trend across varying gene lengths. **B.** *cis- and trans-RSV have opposing effects on TyrRS in hESC-derived neurons.* Human ESC-derived cortical neurons were treated with *cis*- and *trans*-RSV (50 μM) for 8 hr and changes in TyrRS were quantified using IF (n=40 neurons per condition for N=3 independent experiments). Statistical analysis was done using 2way ANOVA with Tukey’s multiple comparisons test. (*p* values *=≤0.05, **=≤0.01, ***=≤0.001, ****=≤0.0001) **C.** *cis-RSV upregulates genes involved in fatty acid degradation.* KEGG pathway enrichment for genes upregulated after treatment with *cis*-RSV. **D.** *Phenylalanine and trans-RSV evoke common DEGs profile including AD-like gene expression signature in KEGG pathway analysis.* Pathway enrichment for KEGG pathways enriched for 866 DEGs common between phenylalanine and *trans*-RSV.
